# TRE5-A retrotransposition profiling reveals putative RNA polymerase III transcription complex binding sites on the *Dictyostelium* extrachromosomal rDNA element

**DOI:** 10.1371/journal.pone.0175729

**Published:** 2017-04-13

**Authors:** Thomas Spaller, Marco Groth, Gernot Glöckner, Thomas Winckler

**Affiliations:** 1 Pharmaceutical Biology, Institute of Pharmacy, University of Jena, Jena, Germany; 2 Core Facility DNA Sequencing, Leibniz Institute for Age Research–Fritz Lipmann Institute, Jena, Germany; 3 Institute for Biochemistry I, Medical Faculty, University of Cologne, Cologne, Germany; Keio University, JAPAN

## Abstract

The amoeba *Dictyostelium discoideum* has a haploid genome in which two thirds of the DNA encodes proteins. Consequently, the space available for selfish mobile elements to expand without excess damage to the host genome is limited. The non-long terminal repeat retrotransposon TRE5-A maintains an active population in the *D*. *discoideum* genome and apparently adapted to this gene-dense environment by targeting positions ~47 bp upstream of tRNA genes that are devoid of protein-coding regions. Because only ~24% of tRNA genes are associated with a TRE5-A element in the reference genome, we evaluated whether TRE5-A retrotransposition is limited to this subset of tRNA genes. We determined that a tagged TRE5-A element (TRE5-A^bsr^) integrated at 384 of 405 tRNA genes, suggesting that expansion of the current natural TRE5-A population is not limited by the availability of targets. We further observed that TRE5-A^bsr^ targets the ribosomal 5S gene on the multicopy extrachromosomal DNA element that carries the ribosomal RNA genes, indicating that TRE5-A integration may extend to the entire RNA polymerase III (Pol III) transcriptome. We determined that both natural TRE5-A and cloned TRE5-A^bsr^ retrotranspose to locations on the extrachromosomal rDNA element that contain tRNA gene-typical A/B box promoter motifs without displaying any other tRNA gene context. Based on previous data suggesting that TRE5-A targets tRNA genes by locating Pol III transcription complexes, we propose that A/B box loci reflect Pol III transcription complex assembly sites that possess a function in the biology of the extrachromosomal rDNA element.

## Introduction

Mobile elements are obligate genomic parasites that amplify as selfish DNA and play important roles in driving the evolution of their hosts [1–5]. Retrotransposons amplify by reverse transcription of RNA intermediates and integration of the resulting DNA copies at new locations of their host’s genomes. Retrotransposons can be distinguished by their overall structures and retrotransposition mechanisms as long terminal repeat (LTR) retrotransposons and non-LTR retrotransposons [6]. Non-LTR retrotransposons probably amplify by target site-primed reverse transcription (TPRT) [7, 8]. In this process, an element-encoded endonuclease cleaves one strand of the genomic DNA to generate a free 3’ hydroxyl that is used as a primer for reverse transcription of the retrotransposon’s RNA by the enzyme reverse transcriptase (RT) [9, 10]. Second-strand cleavage and cDNA joining to genomic DNA usually produce structural hallmarks of integrated retrotransposon copies, such as 5’ deletions, addition of non-templated nucleotides at 5’ insertion junctions, and variable-length target site duplications (TSDs) [11].

In the genome of the soil-dwelling protist *Dictyostelium discoideum*, two thirds of genomic DNA codes for genes, and intergenic regions are mostly less than 1 kb in length with a mean value of 678 bp [12]. The *D*. *discoideum* genome is populated by a family of seven phylogenetically related non-LTR retrotransposons. These elements are named “TRE5” if located ~50 bp upstream of tRNA genes, whereas “TRE3” elements reside ~100 bp downstream of tRNA genes [13, 14]. Sixty-one percent of tRNA genes in the *D*. *discoideum* genome are associated with at least one element of the TRE family. A single tRNA gene may be associated with both TRE5 and TRE3 elements, and TREs that integrate at a tRNA gene previously targeted by another TRE typically form TRE-TRE tandems without integrating into each other.

**Fig 1:**
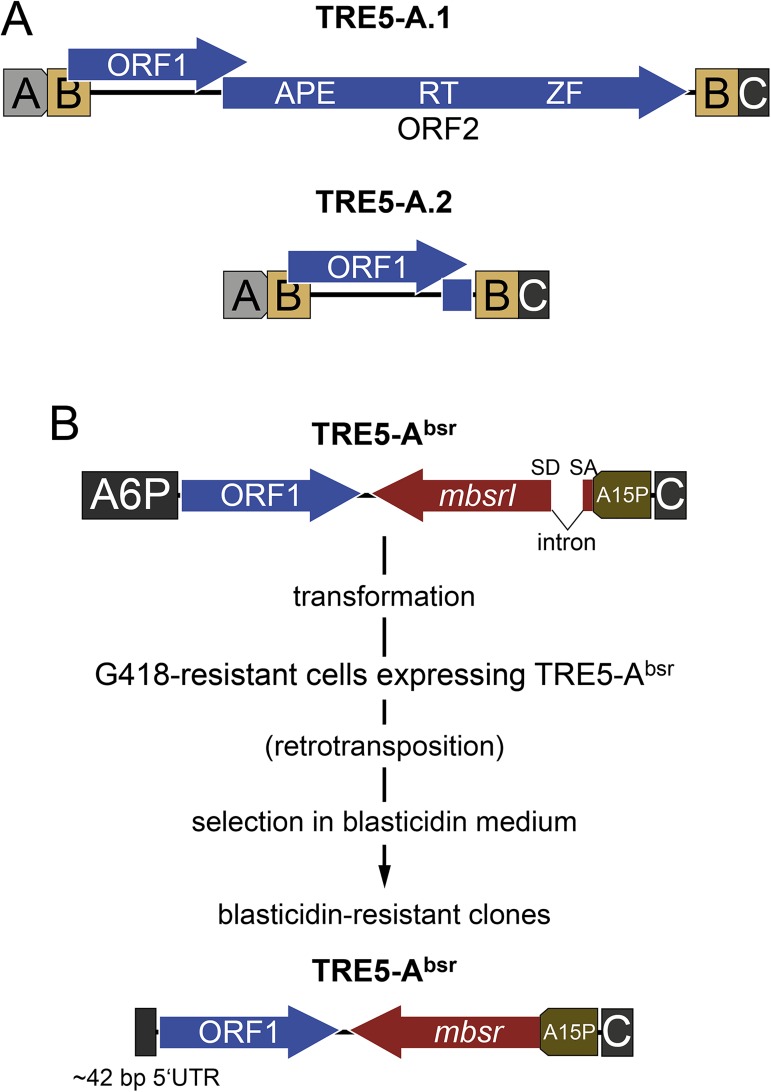
Structures of natural TRE5-A elements and the cloned TRE5-A^bsr^. (**A**) The autonomous retrotransposition-competent TRE5-A.1 element is shown schematically. This element consists of two open reading frames (ORF1 and ORF2) that are flanked by untranslated regions. ORF2 contains an apurinic or apyrimidinic site DNA repair endonuclease (APE), a reverse transcriptase domain (RT), and a zinc finger-like motif (ZF). The A-module has promoter activity for plus strand transcription [20] but can be replaced by a heterologous promoter [21]. The C-module is essential for retrotransposition [21]. The TRE5-A.2 element is a non-autonomous derivative of TRE5-A.1. It is apparently mobilized in trans by the ORF2 protein provided by TRE5-A.1 elements [18]. TRE5-A.2 served as a blueprint for the design of TRE5-A^bsr^ elements. (**B**) Outline of the TRE5-A^bsr^ retrotransposition assay. In the cloned TRE5-A^bsr^ element, the A-module promoter is replaced by the stronger *actin6* promoter (A6P). TRE5-A^bsr^ further contains the ORF1 gene and the C-module of TRE5-A.1. Downstream of ORF1 is the *mbsrI* gene, which is inserted in the retrotransposon’s minus strand direction. The *mbsrI* gene is transcribed from the *actin15* promoter (A15P). The TRE5-A^bsr^ element is transformed into *D*. *discoideum* cells together with a vector that confers G418 resistance to transformants. The transformed TRE5-A^bsr^ is inserted at random positions in the *D*. *discoideum* genome. When the transformed element is transcribed, the intron is removed and restores *mbsr* ORF function after completion of reverse transcription and integration. Cells affected by at least one retrotransposition obtain blasticidin resistance due to the expression of the functional *mbsr* gene. In a typical retrotransposition assay, a pool of G418-resistant cells was subjected to selection in medium containing blasticidin. Apparent clones were counted and pooled for genomic DNA preparation. UTR: untranslated region.

TRE5-A has emerged as a model element to study mechanisms of tRNA gene targeting in the *D*. *discoideum* genome [15]. Full-length TRE5-A.1 contains two open reading frames (ORFs) that are flanked by modularly arranged untranslated regions (Fig 1A). The TRE5-A ORF1 protein does not display similarity with ORF1 proteins of other *D*. *discoideum* TRE elements or non-LTR retrotransposons from other organisms such as mammalian L1. The L1 ORF1 protein is involved in binding the retroelement’s RNA as part of the pre-integration complex and contributes to the integration process [16, 17]. The TRE5-A ORF2 protein contains an apurinic or apyrimidinic site DNA repair endonuclease (APE) and an RT (Fig 1A). TRE5-A.2 is a non-autonomous derivative of TRE5-A.1 that lacks the entire ORF2 sequence. In previous experiments using an in vivo assay that determines the retrotransposition activity of the cellular TRE5-A population, the “TRE trap assay” [18, 19], we determined that TRE5-A.1 and TRE5-A.2 are similarly active, suggesting that TRE5-A.2 efficiently recruits the ORF2 protein delivered by TRE5-A.1 to mediate its retrotransposition.

The A- and B box motifs of tRNA gene-internal control regions represent the binding sites for the Pol III transcription factor TFIIIC [22]. DNA-bound TFIIIC positions TFIIIB near the transcription start, and TFIIIB subsequently recruits Pol III to the 5' ends of tRNA genes to initiate transcription. TRE5-A requires a functional B box for efficient integration upstream of tRNA genes [19]. An isolated B-box with no tRNA gene context is a principle target of TRE5-A integration, albeit at < 1% of the frequency of a canonical tRNA gene [19], suggesting that the presence of an A box may be a second major determinant of integration site selection by TRE5-A. Because the TFIIIB subunit TATA-binding protein (TBP) interacts with TRE5-A ORF1 in vitro [23], we speculate that TFIIIC is required for TRE5-A integration because TFIIIC recruits TFIIIB to the tRNA gene. Then, the ORF1 protein of the TRE5-A pre-integration complex may compete with Pol III for binding to TFIIIB in active Pol III transcription units.

In this study, we used a previously described in vivo retrotransposition assay based on a genetically tagged TRE5-A element (TRE5-A^bsr^; Fig 1B) [21] to determine whether TRE5-A integration is limited to a certain set of tRNA genes in the *D*. *discoideum* genome. We also found that TRE5-A retrotransposes to the extrachromosomal DNA element that carries the ribosomal RNA genes and integrates at the Pol III-transcribed 5S gene and locations that present bona fide A/B-box arrangements without a typical tRNA gene context. Because these sites were targeted as if they were canonical tRNA genes, we speculate that Pol III transcription complexes assemble on the extrachromosomal rDNA element to mediate specific biological functions.

## Materials and methods

### Primers

Oligonucleotides were synthesized at Metabion (Planegg, Germany). A list of primers used in this study is provided in S1 Table.

### *D*. *discoideum* transformation and TRE5-A^bsr^ retrotransposition assay

*D*. *discoideum* AX2 cells were used in all experiments. Construction of TRE5-A^bsr^ and the retrotransposition assay have been described previously [21]. Briefly, *D*. *discoideum* cells were co-transformed with the vectors pTRE5-A^bsr^ and pISAR by electroporation and cultured in HL5 medium (Formedium, Hunstanton, UK) supplemented with 10 μg/ml G418 (Formedium, Hunstanton, UK). G418-resistant clones from several transformations were pooled and maintained as frozen stocks. Cells were re-cultured from the stocks and allowed to grow for approximately 20 or 100 generations in HL5 medium containing 10 μg/ml G418. These cultures are referred to as 20G and 100G, respectively. To collect blasticidin-resistant clones for TRE5-A^bsr^ integration profiling, 60 Petri dishes were seeded with 1 × 10^7^ cells per plate in HL5 medium containing 5 μg/ml blasticidin (Life Technologies, Carlsbad, USA). Blasticidin-resistant clones emerging after 8–10 days were counted, pooled, and used to prepare genomic DNA. In this study, we modified the original TRE5-A^bsr^ by replacing the ORF1 sequence with a codon-adapted version that can be specifically detected by PCR and discriminated from endogenous TRE5-A elements. The codon-adapted ORF1 gene was synthesized by GenScript (Piscataway, USA).

### Preparation of genomic DNA from *D*. *discoideum* cells

*D*. *discoideum* cells were washed in 17 mM phosphate buffer (pH 6.2), and 5 × 10^8^ cells were lysed in 50 ml of buffer A (0.32 M sucrose, 10 mM Tris-HCl, pH 7.5, 5 mM MgCl_2_, 1% Triton X-100). The lysates were kept on ice for 10 min before the nuclei were pelleted by centrifugation at 4°C. The nuclear pellets were resuspended in 5 ml of buffer B (10 mM Tris-HCl, pH 7.5, 10 mM EDTA), mixed with 5.5 ml of buffer C (10 mM Tris-HCl, pH 7.5, 0.7% SDS) and 30 μg of RNase A (Sigma), and incubated at 37°C. After 30 min, 800 μg of proteinase K (Fermentas) was added, and incubation was continued at 55°C for 60 min. DNA was extracted once in 10 ml of a 25:24:1 mixture of phenol, chloroform, and isoamyl alcohol and once in 8 ml of a 14:1 mixture of chloroform and isoamyl alcohol. The DNA was precipitated in ethanol overnight at –20°C, washed with 70% ethanol, dried, and suspended in 400 μl of water. After the DNA had dissolved, the ethanol precipitation was repeated, and the pelleted DNA was washed three times in 70% ethanol. Finally, the dry DNA was recovered in 10 mM Tris-HCl (pH 7.5) and 1 mM EDTA and stored at 4°C.

### Enrichment of TRE5-A^bsr^ integrations by LAM-PCR

Linear amplification was performed in a 50-μl volume containing 2 μg of genomic DNA and 0.5 pmol of a 5'-biotinylated TRE5-A^bsr^ ORF1-specific primer (Rep-234_bio) using the High-Fidelity Polymerase Kit from Jena Bioscience (Jena, Germany). A typical reaction was conducted with 100 cycles of annealing at 58°C for 30 sec and elongation at 68°C for 45 sec. Biotinylated single-stranded PCR products were purified using the QIAquick PCR Purification Kit (Qiagen, Germany) and then immobilized onto streptavidin-conjugated magnetic beads (Dynabeads^®^ M-280, Invitrogen) to remove contaminating genomic DNA. To prepare the “tDNA primer library”, exponential parallel PCR was performed on the bead-bound single-stranded LAM-PCR products with a set of distinct reverse primers specific for particular tRNA gene families. The PCR amplimers were purified in agarose gels, pooled, and subjected to Illumina amplicon sequencing. To prepare the “adapter primer library”, bead-bound single-stranded LAM-PCR products were subjected to second-strand DNA synthesis using an adapter-random hexamer primer (454B-6N) and Klenow polymerase at 37°C for 60 minutes. Because LAM-PCR does not discriminate between transformed and retrotransposed TRE5-A^bsr^ elements, the double-stranded LAM-PCR products were digested after second-strand DNA synthesis with NcoI, which cuts the plasmid immediately upstream of the *act6* promoter of the cloned TRE5-A^bsr^ element. DNA from individual PCR reactions was gel-purified (QIAquick Gel Extraction Kit, Qiagen) and either cloned into pGEM-T (Promega) for manual DNA sequencing or pooled for paired-end Illumina sequencing.

### Illumina amplicon sequencing

Amplicons of the 20G tDNA, 100G tDNA, and 100G adapter primer libraries, prepared as described above, were sequenced using the Illumina technique [24]. The concentration of amplicons was determined using the Agilent Bioanalyzer 2100 and DNA 7500 kit. Library preparation was performed using Illumina's TruSeq® DNA PCR-Free LT Sample Preparation Kit following the manufacturer's description, except that amplicons were not fragmented before library preparation. 100 ng of amplicon DNA were used as input material, and the protocol was started at the step of end-repair. Libraries were individually indexed as part of the protocol and quantified/qualified using the Agilent Bioanalyzer 2100 as described above. Samples were sequenced on Illumina's MiSeq in 300 bp paired-end mode using sequencing chemistry MiSeq® Reagent Kit v3 (600 cycle). The reads were extracted in FastQ format by the MiSeq control software (MCS v2.4.1.3) or bcl2fastq v2.16/v2.17.1 software (Illumina). The raw data (fastq files) of Illumina amplicon sequencing are available at the NCBI Short Read Archive (SRA) (http://www.ncbi.nlm.nih.gov/sra/) under accession number SRP098685.

### Mapping of tRNA gene-targeted TRE5-A^bsr^ integrations

To determine TRE5-A^bsr^ insertion sites in the *D*. *discoideum* genome, reads were pre-processed to extract the genomic-derived part of the amplicons for the later mapping approach. In the first step, “pseudoreads” were created by joining read 1 (forward read) and read 2 (reverse read) of each pair using clc_overlap_reads (part of the CLC assembly cell, CLCbio) with parameter–m 100 (discard pseudoreads with lengths <100nt). The reads were re-formatted into FastA format for the later mapping process. Only for analysis of the adapter primer library, pseudoreads were discarded from the dataset before mapping if they did not contain the Rep-235 sequence in order to remove artificial constructs.

In the next step, the pseudoreads were mapped to the TRE5-A^bsr^ sequence using SSAHA [25] to determine the TRE5-A^bsr^-derived part of the reads. Based on this mapping the retrotransposon-derived part of the reads was trimmed using an in-house script. To trim the tRNA gene part of the reads in the 20G and 100G tDNA primer libraries, the retrotransposon-free reads were mapped to a reference dataset containing all *Dictyostelium* tRNA gene families (extracted from the file “*D*. *discoideum* Non-coding sequences”, downloadable at http://dictybase.org) using SSHA and were trimmed based on the mapping results. Since in the adapter primer library a random hexamer was used for amplification, the second trimming was performed based on the adapter sequence instead of the tRNA reference.

After trimming of retrotransposon and tRNA/adapter parts, pseudoreads were discarded if shorter than 40 nt in length in order to avoid imprecise mapping results by multiple mappable short pseudoreads. To define the insertion sites, the pre-processed reads (pseudoreads, retrotransposon and tRNA/adapter part trimmed) were mapped to the *Dictyostelium* reference genome (version 05-13-2009; http://dictybase.org) using BLAT [26]. In addition, pseudoreads were mapped to the left arm of the rDNA palindrome as published in GenBank AY171066 [27]. To avoid false positive results we only allowed following hits: (i) unique hits, and (ii) reads with multiple hits when fulfilling the following criteria: Score of the 2^nd^-best hit should not exceed 75% of the score of the best hit; the read must map with at least 95% of its length. The mapping results were manually inspected for TRE5-A^bsr^ insertions at 405 tRNA gene positions and on the extrachromosomal rDNA palindrome using the Integrative Genomics Viewer (version 2.3.65) [28].

### Reverse-transcription PCR on circularized RNA (cRT-PCR)

The RNA samples for RT-PCR were prepared from logarithmically growing *D*. *discoideum* cells washed in 17 mM phosphate buffer (pH 6.2) and stored as pellets of 2 × 10^7^ cells at –80°C until further use. Total RNA was prepared from frozen cells using the Qiagen RNeasy^®^ Mini Kit according to the provided manual. cDNA was synthesized by the reverse transcription of 500 ng of total RNA using the Qiagen Omniscript^®^ RT Kit. RT-PCR on circularized RNA (cRT-PCR) was performed as previously described [29]. Briefly, the total RNA was 5'-dephosphorylated with shrimp alkaline phosphatase (Fermentas) and then re-phosphorylated using Fermentas T4 polynucleotide kinase. The phosphorylated RNA was then self-ligated using T4 RNA ligase (Fermentas). Reverse transcription was performed using the primer cRT-PCR-01 and the Qiagen Omniscript^®^ RT Kit. The first PCR was conducted with the primers cRT-PCR-02 and cRT-PCR-03 using the following cycling conditions: 95°C for 5 min and 35 cycles of 95°C for 30 sec, 58°C for 30 sec, and 72°C for 45 sec. The PCR products were purified using Qiagen's PCR Purification Kit, followed by a nested PCR using the same cycling conditions and the primers cRT-PCR-04 and cRT-PCR-05. The sequences of the cRT-PCR primers for each locus are listed in (S1 Table). The cRT-PCR products were analyzed on agarose gels, cloned into pGEM-T (Promega) and sequenced.

## Results

### TRE5-A^bsr^ mimics the integration behavior of the natural TRE5-A population

A characteristic feature of the *D*. *discoideum* genome is the association of tRNA genes with retrotransposons of the TRE family. The annotated genome features 42 tRNA gene families comprising 405 nuclear tRNA genes scattered on all six chromosomes (S1 Fig). Although natural TRE5-A integrations cover 31 of 42 tRNA gene families in the *D*. *discoideum* reference genome, only 24% of individual tRNA genes are associated with a TRE5-A. This raised the question as to whether the natural TRE5-A population is restricted to this particular set of targets. To address this question, we transformed the TRE5-A^bsr^ element into *D*. *discoideum* cells and performed a genome-wide survey of TRE5-A^bsr^ integration sites. The TRE5-A^bsr^ element is a non-autonomous TRE5-A.2-like element that contains the ORF1 gene and an *mbsrI* gene as a selection marker for productive retrotransposition (Fig 1B). The *mbsrI* gene is a blasticidin resistance gene (*bsr*) cloned into a minimal TRE5-A in the minus strand orientation (= *mbsr*). The *mbsr* gene is initially not functional because it is interrupted by a reverse intron (= *mbsrI*). After retrotransposition, the newly integrated TRE5-A^bsr^ copy contains a functional *mbsr* gene that confers blasticidin resistance to the affected cells (Fig 1B). In the experiments described below, the original ORF1 sequence of the TRE5-A^bsr^ element was replaced by a codon-adapted ORF1 sequence that allowed us to discriminate TRE5-A^bsr^ elements from cellular TRE5-As. Similar to the non-autonomous TRE5-A.2 element, TRE5-A^bsr^ is readily mobilized in trans by the ORF2 protein provided by the cellular population of full-length TRE5-A.1 elements [21].

**Fig 2:**
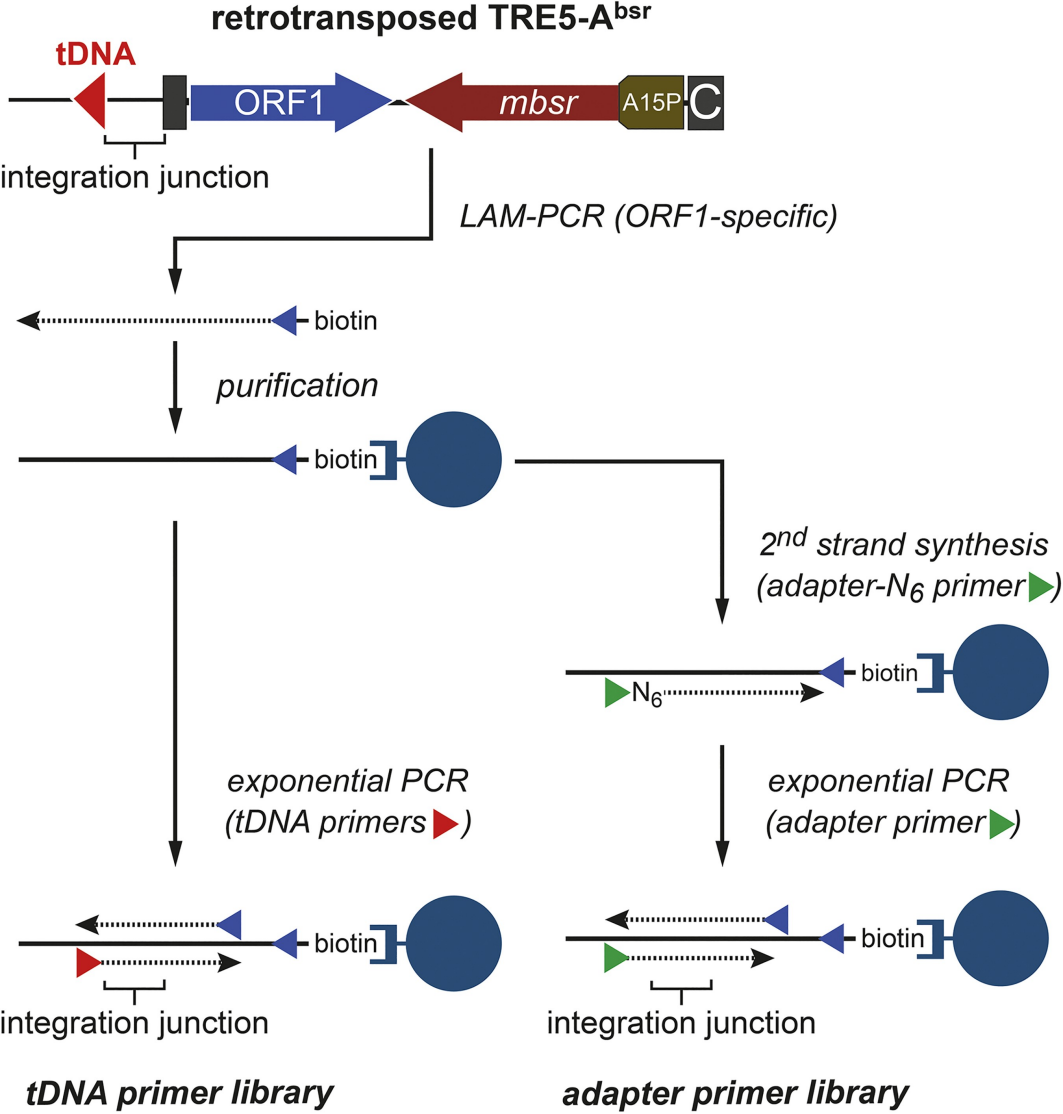
Outline of TRE5-A^bsr^ retrotransposition profiling. Note that TRE5-A always integrates in an orientation-specific manner with the 5’ end of the retrotransposon facing the 5’ end of the targeted tRNA gene. LAM-PCR was performed on genomic DNA prepared from a pool of blasticidin-resistant clones. The 5'-biotinylated primer bound selectively to the codon-adapted ORF1 gene of the TRE5-A^bsr^ element and did not recognize ORF1 genes of endogenous TRE5-A elements. The resulting linear, single-stranded LAM-PCR products were immobilized on streptavidin beads and washed extensively. To perform profiling of TRE5-A^bsr^ insertions at tRNA genes, exponential PCRs were performed in parallel reactions with primers specific for selected tRNA gene families (“tDNA primer library”). To profile TRE5-A^bsr^ insertions at any position in the genome, second-strand synthesis was initiated with a random hexamer primer linked to a unique adapter oligonucleotide (adapter-N_6_). Next, exponential PCR was performed with the adapter primer and an ORF1-specific primer to yield an “adapter primer library”. SD, splice donor site; SA, splice acceptor site.

In a typical TRE5-A retrotransposition profiling experiment, *D*. *discoideum* cells expressing the TRE5-A^bsr^ element were grown under G418 selection for approximately 20 generations (referred to as “20G culture”). Then, a total of 6 × 10^8^ cells was subjected to blasticidin selection, which enriched cells in which TRE5-A^bsr^ had completed at least one full retrotransposition event. Approximately 15,000 blasticidin-resistant clones were recovered, pooled, and used for genomic DNA preparation. Based on this pool we wanted to apply a transposon profiling based on Illumina amplicon sequencing of TRE5-A^bsr^ integration junctions to determine how many tRNA genes were targeted by TRE5-A^bsr^ integration in the 20G culture. We decided to use non-restrictive linear amplification PCR (LAM-PCR), because alternative methods, including inverse PCR [30], ligation of adapter linkers to digested genomic DNA (ligation-mediated PCR; [31]), or ligation of single-stranded linkers to the 3’ ends of single-stranded LAM-PCR products [32], did not produce high-quality libraries for amplicon sequencing of tDNA-TRE5-A^bsr^ integration junctions. TRE5-A^bsr^ DNA was enriched using LAM-PCR as shown in Fig 2. Genomic DNA was used as the template for linear PCR with a biotinylated primer that bound specifically to the codon-adapted ORF1 sequence in the cloned TRE5-A^bsr^ element. The linear, single-stranded LAM-PCR products were immobilized on magnetic streptavidin beads and washed extensively to remove unrelated genomic DNA, including endogenous TRE5-A sequences (S2 Fig).

Integration of TRE5-A is strictly orientation-specific, with the 5’ end of the element always facing the 5’ end of the targeted tRNA gene. We took advantage of this integration behavior to specifically screen for TRE5-A^bsr^ integration at tRNA genes in parallel exponential on-bead PCRs with a TRE5-A^bsr^ ORF1-specific forward primer and a set of reverse primers intended to cover 33 selected tRNA gene families. Due to apparent cross-hybridization of some primers among related tRNA gene families, the analysis actually covered 37 of the 42 tRNA gene families and 398 of the 405 individual tRNA genes (see below). The copy numbers of individual tRNA gene families in the *D*. *discoideum* genome vary between 1 and 23 (S1 Fig). Therefore, the obtained PCR bands in agarose gels contained mixtures of PCR products representing integrations at individual chromosomal loci of the same tRNA gene family.

**Fig 3:**
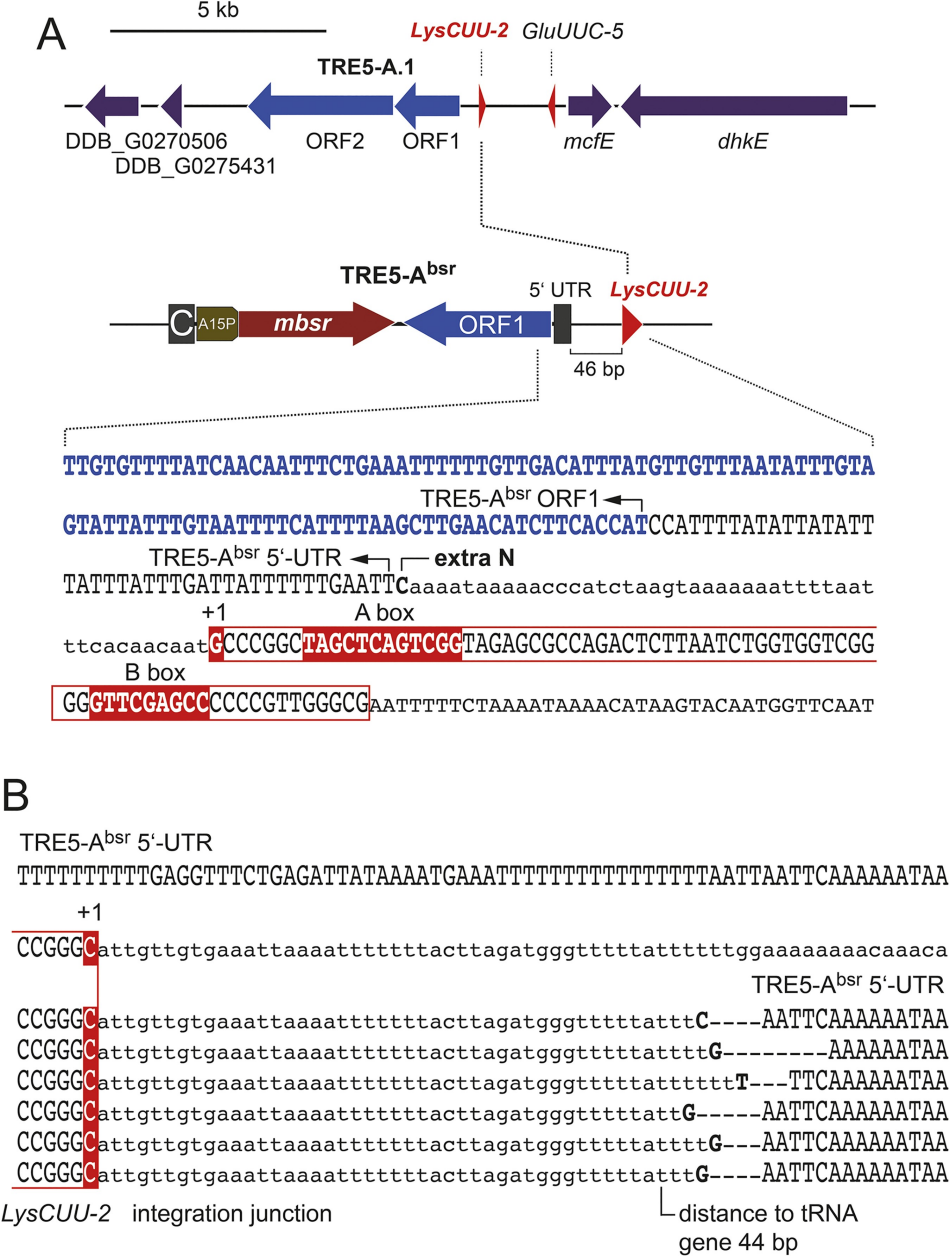
TRE5-A^bsr^ integrations at the *LysCUU-2* gene. (**A**) Gene map around the *LysCUU-2* gene (coordinates 2688000 to 2706000 on chromosome 1). Note that the gene is associated with a full-length TRE5-A.1 element, of which only the annotated ORF1 and ORF1 regions are shown. In the sequenced example shown, TRE5-A^bsr^ integrated 46 bp upstream of the *LysCUU-2* gene, thus forming a tandem with the endogenous TRE5-A.1 element. Note that TRE5-A^bsr^ left most of the *act6* promoter behind, and the retrotransposed copy contained 42 bp of the 5' untranslated region (5'-UTR) upstream of the ORF1 gene. The tRNA gene sequence is boxed. The +1 G nucleotide, A box, and B box are shown with a red background. A non-template extra C nucleotide is indicated, and the integration junction upstream of the +1 G is shown in lowercase letters. “DDB_G” refers to gene ID numbers listed in dictyBase [34]. (**B**) Individual integrations of TRE5-A^bsr^ were cloned from PCR products obtained with a *LysCUU*-specific and TRE5-A^bsr^ ORF1-specific primer. The upper lane shows the sequence of the TRE5-A^bsr^ 5’-UTR (i.e., the *act6* promoter) in the transformed TRE5-A^bsr^ element. Below is the sequence upstream of the *LysCUU-2* gene (upstream sequence in lowercase letters). The sequences of six integration junctions are shown with the +1 G complement of the *LysCUU2* with a red background. The integration junction sequence is shown in lowercase letters. Note the insertion of non-templated extra-nucleotides (bold uppercase letters), which help distinguish insertions that occurred at exactly the same position.

To confirm the quality of PCR products representing tRNA gene-specifically amplified TRE5-A^bsr^ integration junctions, we cloned and sequenced the PCR products from several tRNA gene families. As an example, Fig 3 shows the results for *LysCUU*, of which 10 gene copies exist in the *D*. *discoideum* genome. The *LysCUU-2* gene is located on chromosome 1 and is associated with an endogenous, full-length TRE5-A.1 element. A new integration of TRE5-A^bsr^ was observed 46 bp upstream of the *LysCUU-2* gene (Fig 3A), suggesting the formation of a TRE5-A^bsr^-TRE5-A.1 tandem. As expected, the heterologous RNA polymerase II-transcribed *actin6* promoter of TRE5-A^bsr^ was left behind upon retrotransposition, resulting in a ~42-bp 5'-untranslated region in the retrotransposed TRE5-A^bsr^ coinciding with the experimentally determined transcription start site of the *actin6* gene [33]. The integration junctions of six retrotransposed TRE5-A^bsr^ copies at the *LysCUU-2* gene could be distinguished due to different distances between the integrated element and the target; integrations occurred within a window of 44–48 bp upstream of the targeted tRNA gene (Fig 3B). The precision of TRE5-A^bsr^ integration complicated the discrimination of individual retrotransposition events at the same genomic locus because individual integrations at exactly the same position upstream of the targeted tRNA gene could only be distinguished by short deletions of the 5’ ends of integrated TRE5-A^bsr^ copies and/or the addition of non-templated nucleotides at the insertion junctions introduced by TRE5-A RT during the integration process (Fig 3B).

**Fig 4:**
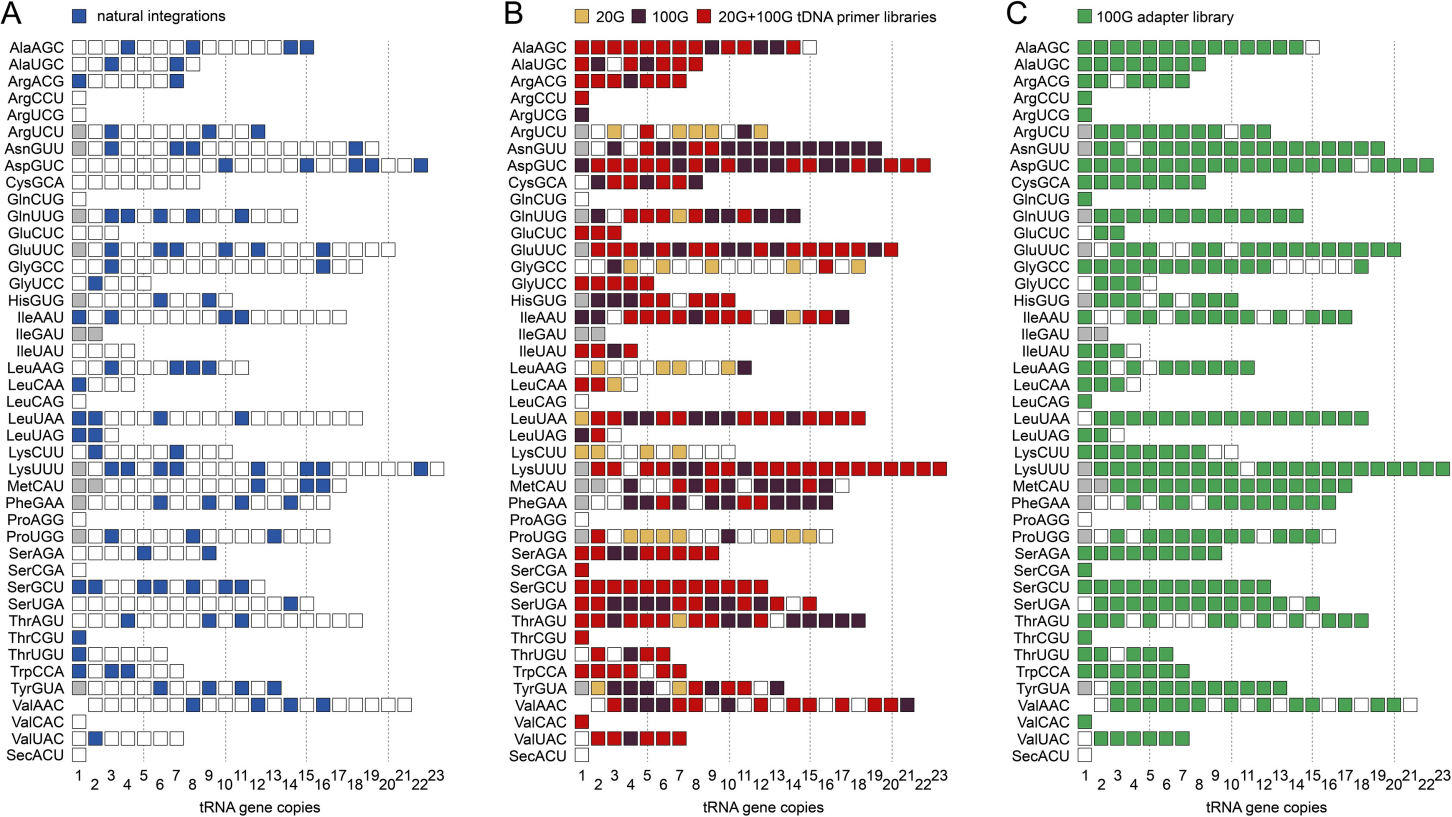
Visualization of TRE5-A^bsr^ integrations at tRNA genes. The 42 tRNA gene families and their individual copy numbers are shown as white squares. The colors indicate the association of a distinct tRNA gene copy with a TRE5-A or TRE5-A^bsr^ element. See S1 Fig for the distribution of individual tRNA genes on the six chromosomes. Gray squares indicate mitochondrial tRNA genes. (**A**) Display of tRNA gene loci associated with a TRE5-A element. (**B**) TRE5-A^bsr^-targeted tRNA genes identified in the tDNA primer library prepared after 20 generations (20G) or 100 generations (100G) of cell culture. Light red and dark red squares indicate tRNA genes identified only in the 20G or 100G library, respectively. Bright red squares indicate tRNA genes found in both libraries. (**C**) TRE5-A^bsr^-targeted tRNA genes found in the adapter primer library prepared after 100 generations of cell culture (100G).

In the *D*. *discoideum* reference genome, only 98 of 405 tRNA genes are associated with a TRE5-A element (Fig 4A). This limitation may be due to a general inaccessibility of the majority of tRNA genes to TRE5-A integration. Alternatively, multicopy association of TRE5-A with tRNA could pose a selective disadvantage for the organism that resulted in a steady-state at 24% saturation. To assess whether TRE5-A retrotransposition is restricted to only a subset of tRNA genes, we used TRE5-A^bsr^-enriched LAM-PCR products of the 20G culture respresenting ~15,000 blastidicin-resistant clones to perform exponential PCR with a set of 33 tRNA gene families. The PCR amplicons were gel-purified and pooled to generate a “tDNA primer library” (Fig 2). We performed Illumina paired-end sequencing on this DNA and assembled matching read pairs into “pseudoreads” of forward and reverse sequence reads. These pseudoreads were searched for sequences covering TRE5-A^bsr^ upstream of the primer used for exponential PCR and for the presence of the sequences of tRNA gene-specific primers at the other end of the pseudoreads. Following this routine, a dataset of 235,729 pseudoreads was generated (Table 1). We found that 206,652 mapped to the genome, of which 205,020 could be assigned to 223 individual tRNA gene positions (Fig 4B and S2 Table). The de novo TRE5-A^bsr^ integrations covered 91 of 98 tRNA genes already occupied by a copy of the natural TRE5-A population, suggesting that TRE5-A^bsr^-TRE5-A tandems were formed upstream of these tRNA genes. We identified TRE5-A^bsr^ integrations at 132 tRNA genes not occupied by natural TRE5-A, indicating that the integration spectrum of TRE5-A is considerably broader than currently evident in the *D*. *discoideum* reference genome. We observed a great variety of pseudoread counts mapping to individual tRNA genes. The mapping counts ranged from 1 to 43,221 per locus (S2 Table). Assuming an average distribution of 506 mapped pseudoreads at 405 targets (= 205,020 pseudoreads), pseudoread numbers mapped to individual loci were highly biased. In fact, only 30 of 223 integration sites were covered by higher pseudoread numbers than the expected average, but these 30 loci accounted for 97% of all mapped pseudoreads. As described later, these apparent “hot spots” of TRE5-A^bsr^ integration did not reproduce in a second integrome library, suggesting that some PCR bias or clonal expansion of cells undergoing retrotransposition events early in the cell culture may be responsible for this observation.

**Table 1:** Summary of Illumina sequence mapping results.

	20G tDNA primer library	100G tDNA primer library	100G adapter primer library
Read pairs	414,722	1,096,059	3,645,898
Assembled pseudoreads	380,745	903,394	1,821,217
Mapped to TRE5-A^bsr^	360,538	865,522	1,102,203
Mapped to tRNA genes/adapter primer	236,410	718,095	1,020,159
Pseudoreads ≥ 40 bp^1^	235,729	710,163	755,593
Mapped to reference genome	206,652	595,188	471,921
Mapped to tRNA genes	205,020	594,367	318,376
Mapped to *actin6* promoter^2^	181	71	68,081
Mapped to rDNA palindrome	n/a	n/a	1,186
Remaining	1,451	750	84,278

^1^ Minimum length of 40 bp was selected to avoid imprecise mapping results in highly A+T-rich integenic regions.

^2^ Mapped to either the *actin6* promoter sequence or the ~42 bp 5’ untranslated region carried along by retrotransposed TRE5-A^bsr^ (see Fig 1B).

Roughly 50% of tRNA genes were identified as TRE5-A^bsr^ targets in the experiment described above, even though 15,000 clones were supposed to provide roughly 37× coverage of theoretically available integration sites (i.e., tRNA genes). We presumed that a limitation in discovering rare integrations at particular targets might be overcome by extending the cultivation time in G418 medium before initiating blasticidin selection, thus allowing TRE5-A^bsr^ retrotransposition to approach saturation at tRNA gene targets. Therefore, we repeated the experiment described above with the same stock of G418-resistant cells but allowed growth of cells under G418 selection for approximately 100 generations before starting blasticidin selection to enrich cells with TRE5-A^bsr^ integrations (referred to as “100G culture”). Approximately 75,000 blasticidin-resistant clones were recovered from 6 × 10^8^ cells, that is, five times more than from the 20G culture, suggesting ongoing retrotransposition in the population upon cultivation under G418 selection. The blasticidin-resistant clones from the 100G culture were pooled, and TRE5-A^bsr^ integrants were enriched by LAM-PCR. Again, parallel exponential PCR was performed on the LAM-PCR products using different tRNA gene family-specific primers. All purified PCR products were mixed and analyzed by Illumina sequencing (Table 1), and the assembled pseudoreads were mapped to the reference genome. In this data set, 594,367 pseudoreads mapped to 303 tRNA gene loci including 83 that are associated with a TRE5-A of the natural population (Fig 4B and S2 Table). As in the first experiment, we observed a bias for the mapping of integrations to particular tRNA gene loci ranging from 1 to 81,177. In contrast to the expectation of an average of 1,468 mappable pseudoreads per locus upon equal selection of 405 potential target genes, 72 tRNA mapped loci presented count numbers above the average and accounted for 91% of all mappable reads. However, only ten of these loci were also overrepresented in the 20G library, suggesting that the numbers of mapped pseudoreads do not correlate with the frequency of targeting events at particular loci. Rather, a technical bias may have led to enrichments of these loci. Nevertheless, the combined data from the 20G and 100G libraries identified 334 (83%) of all tRNA gene positions in the *D*. *discoideum* genome as potential TRE5-A integration sites (Fig 4B).

**Fig 5:**
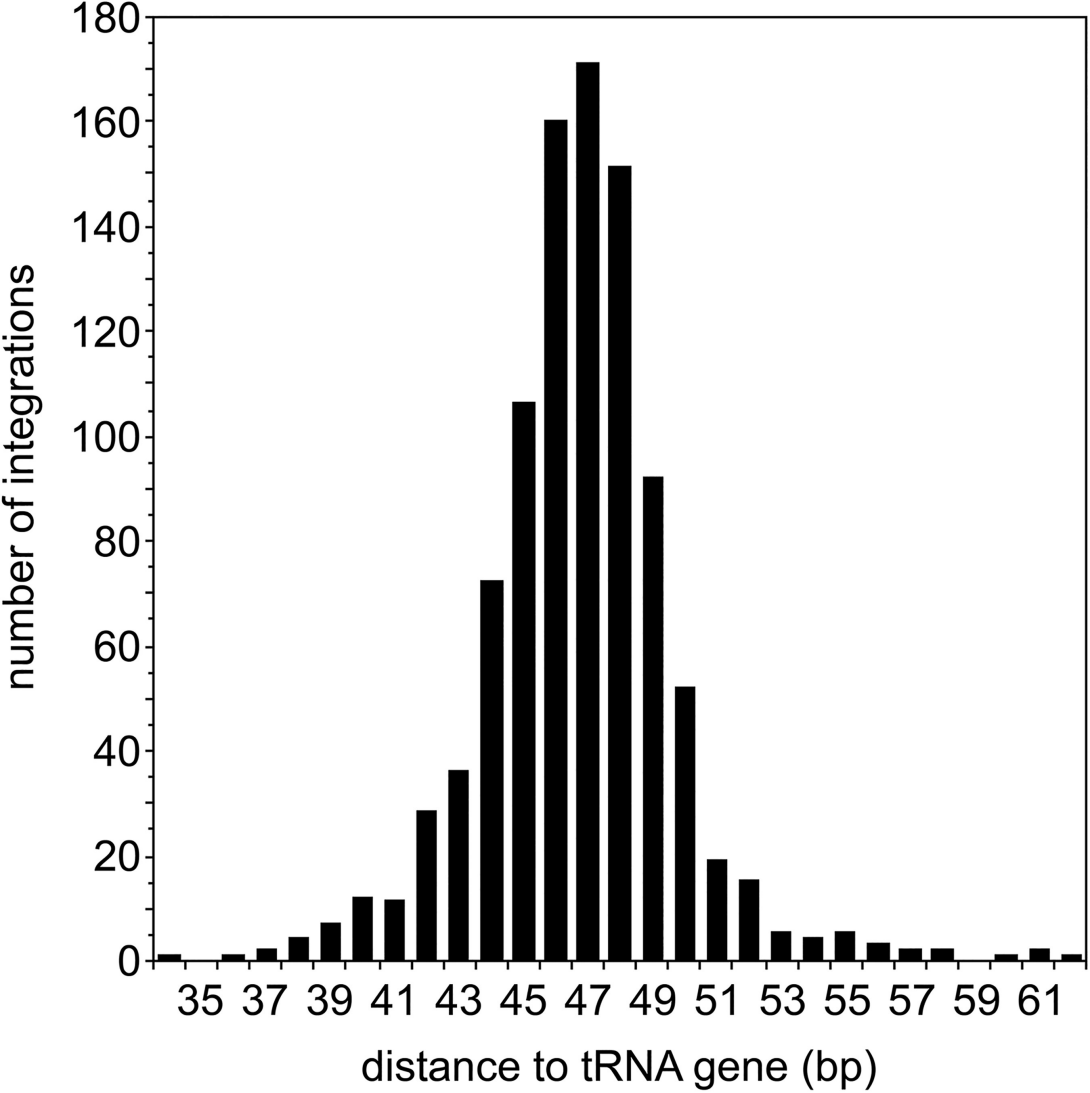
Distances of TRE5-A^bsr^ integrations to targeted tRNA genes. The analysis is based on 303 targeted tRNA gene locations and 1,003 TRE5-A^bsr^ individual integrations that could be distinguished due to different distances to the inserted TRE5-A^bsr^ element to the target. Note that 38 of 1,003 integrations were detected outside the window of 34–62 bp shown here.

We previously determined that the presence of a functional B box is a major determinant for TRE5-A integration at tRNA genes [19]. However, the variable distances of B-boxes to the 5’ ends of tRNA genes do not sufficiently explain the precision of TRE5-A integration ~47 bp upstream of tRNA genes in the reference genome. Because the distances between A- and B-boxes in *D*. *discoideum* tRNA genes vary between 32 and 57 bp, we assumed that the fixed position of the A-box at position +8 relative to the mature tRNA determines the distance of TRE5-A to targeted tRNA genes. The integration preference of TRE5-A^bsr^ relative to the +1 nucleotide of a tRNA gene was determined based on 303 targeted tRNA gene loci in the 100G tDNA library. In many cases, the high precision of integration prevented accurate counting of independent integration events at a given tRNA gene locus when they occurred at exactly the same position. Of 1,003 integrations that could be discriminated by different distances to the same tRNA genes, integration occurred between 23–181 bp upstream of tRNA genes, with 80.2% located within a window of 47±3 bp upstream of the targeted tRNA genes (Fig 5).

In summary, we conclude from our observations that the TRE5-A^bsr^ element is readily mobilized in trans by recruiting the ORF2 protein expressed from the natural TRE5-A.1 population and that TRE5-A^bsr^ faithfully mimics the integration behavior of the natural TRE5-A in *D*. *discoideum* cells.

We were also interested to determine if TRE5-A integrations can occur at genomic locations not represented by tRNA genes. Therefore, we used the pool of blasticidin-resistant cells from the 100G culture to perform LAM-PCR enrichment of TRE5-A^bsr^ integrations. Second-strand synthesis was perfomred using a random hexamer oligonucleotide linked to a unique adapter. We performed exponential PCR on these double-stranded DNA fragments using the adapter primer and a reverse primer specific for the TRE5-A^bsr^ ORF1 sequence, thus including integration sites without tRNA gene context (“adapter primer library”; Fig 2). The amplicons were analyzed by Illumina sequencing as described previously. In this experiment, 755,593 pseudoreads were assembled (Table 1), of which 68,081 mapped to the *actin6* promoter upstream of the ~42-bp 5'-untranslated region. We could not determine if these pseudoreads belonged to contaminating genomic DNA derived from either the chromosomal *actin6* upstream region or the transformed TRE5-A^bsr^ element, which was transcribed from the *actin6* promoter (see Fig 1B). These reads were not further analyzed. A set of 84,278 pseudoreads seemed to map to genomic regions both in open reading frames and in intergenic regions unrelated to Pol III transcription sites. Manual inspection of several such pseudoread sequences revealed complex rearrangements between the *actin6* promoter region of the TRE5-A^bsr^ element and genomic DNA, which may have occurred either during LAM-PCR or during the exponential PCR to prepare the adapter primer library. We attempted to directly detect predicted TRE5-A^bsr^ insertions within ORFs or in intergenic regions by performing PCR on genomic DNA of the 100G library with locus- and TRE5-A^bsr^-specific primers. None of these PCR experiments confirmed the presence of integrated TRE5-A^bsr^. Therefore, we concluded that pseudoreads with signs of recombinations of TRE5-A^bsr^ upstream of the transcription start of the element represented mainly PCR artifacts and did not merit further analysis.

A total of 318,376 pseudoreads of the adapter primer library mapped to 343 tRNA gene positions (Fig 4C, S2 Table). Notably, 93% of the tRNA genes identified as TRE5-A^bsr^ integration targets in the 100G tDNA primer library were also found in the 100G adapter primer library. As in the experiments using tDNA primer libraries, we observed enrichment of pseudoread counts on a small subgroup of targeted tRNA genes. In fact, 30 tRNA genes were mapped with read counts above the statistical average of 928 per site and accounted for 89% of total reads. Of these tRNA genes, only 20 were also overrepresented in the 100G tDNA primer library prepared from the same cell culture. Interestingly, 41 tRNA genes not determined as targets of TRE5-A^bsr^ in the tDNA primer libraries were identified in the 100G adapter primer library, extending the list of TRE5-A^bsr^ integration targets in all experiments to 384 or 95% of all tRNA genes in the *D*. *discoideum* genome.

### TRE5-A^bsr^ integrations on the extrachromosomal rDNA element

*D*. *discoideum* cells organize their ribosomal RNA genes on a multicopy, extrachromosomal DNA element that consists of two identical arms spanning ~44 kb each [27]. Despite the rRNA genes, no other transcription units seem to exist on this element, which amplifies to approximalety 100 copies per cell and accounts for 20% of nuclear DNA. Because of its multicopy nature, the extrachromosomal DNA element presents approximately 200 ribosomal 5S genes per cell as potential target sites for TRE5-A. Therefore, it was of interest to investigate whether TRE5-A is able to target this typical Pol III-transcribed gene. Previous in vivo retrotransposition experiments in which the ribosomal 5S gene was cloned into the artificial chromosomal environment of the “TRE trap” revealed that the natural TRE5-A population can target the upstream region of the 5S gene at a distance of 35–39 bp and at approximately 5% of the frequency of a tRNA gene [19]. In the Illumina sequencing data of the adapter primer library, we determined that 944 pseudoreads mapped to the 5S gene locus on the extrachromosomal rDNA palindrome. Manual inspection of the mapping data revealed that TRE5-A^bsr^ had targeted the upstream region at a distance of 37–41 bp to the 5’ end of the 5S gene. To further evaluate TRE5-A^bsr^ insertions upstream of the 5S gene, we performed exponential PCR on LAM-PCR products using a 5S gene-specific and a TRE5-A^bsr^-specific primer. A PCR product was obtained and cloned (data not shown). Sequencing of the cloned integration junction confirmed TRE5-A^bsr^ integration 38 bp upstream of the 5S gene (S3 Fig). In parallel experiments, we explored whether natural TRE5-A can target the extrachromosomal palindrome upstream of the 5S gene. We performed PCR on genomic DNA of untransformed *D*. *discoideum* cells using a TRE5-A- and a 5S gene-specific primer. A PCR product was obtained and cloned (data not shown). Sequencing of this PCR product confirmed the insertion of a TRE5-A.1 element 38 bp upstream of the 5S gene (S4 Fig). Thus, we concluded that TRE5-A integration is not restricted to tRNA genes and also includes the 5S gene on the extrachromosomal rDNA palindrome.

**Fig 6:**
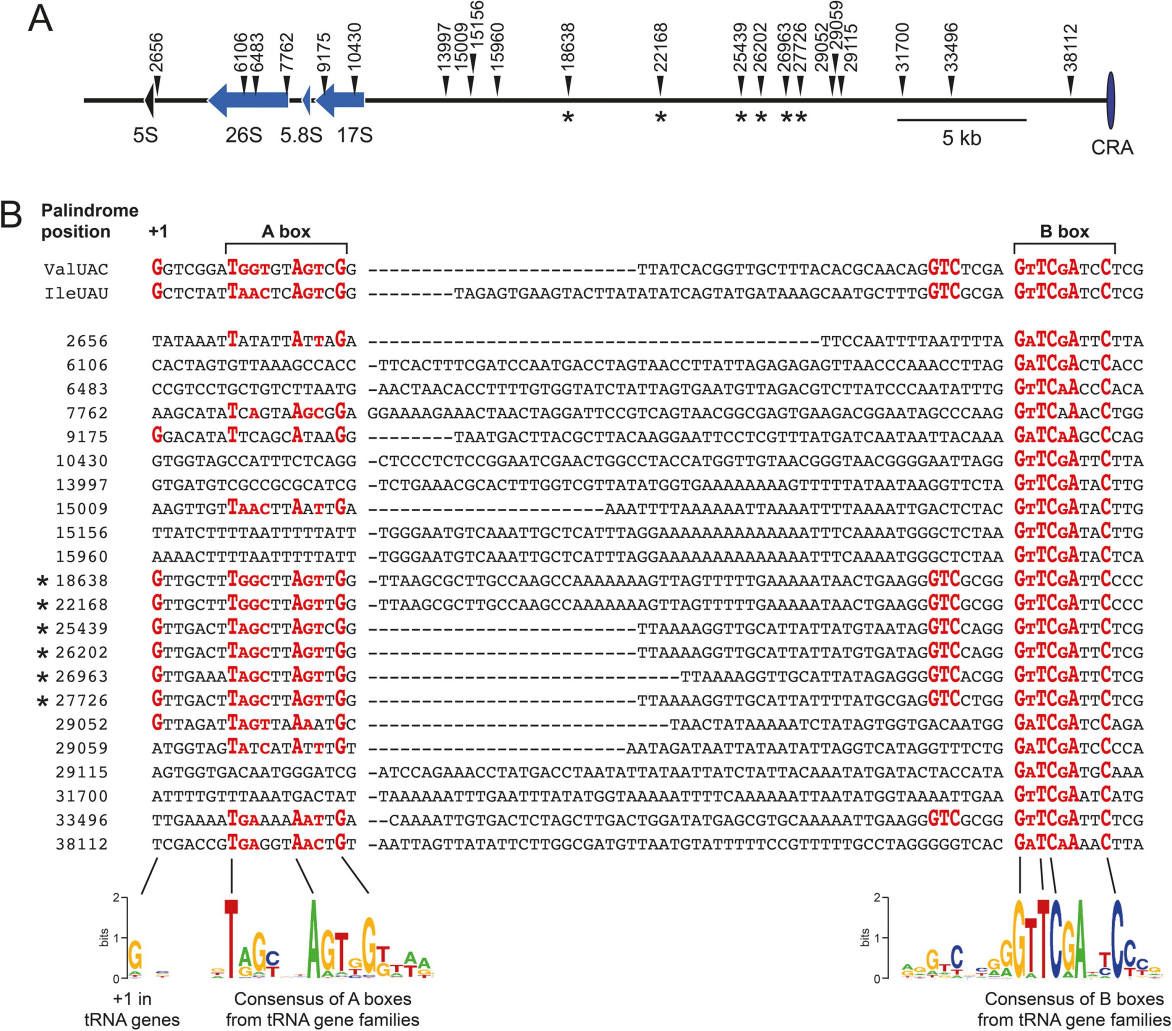
B-boxes on the extrachromosomal rDNA palindrome. (**A**) Scheme of the left arm (~39 kb) of the mirror-symmetric rDNA element. The rRNA genes are indicated as arrows. The B box positions are indicated by numbers according to the rDNA palindrome core sequence as published in GenBank entry AY171066. CRA: central region of asymmetry. The asterisks indicate the TRE5-A^bsr^ integration targets determined in this study. (**B**) Sequences of individual B box loci on the *D*. *discoideum* rDNA palindrome. WebLogo presentations of A box and B box motifs of *D*. *discoideum* tRNA genes are provided for comparison with the respective sequences on the rDNA palindrome. The most conserved nucleotide positions in the B boxes and putative A boxes are highlighted in red. The asterisks indicate the positions where TRE5-A^bsr^ integrations were identified in this study.

In a previous study, we observed by chance that TRE5-A^bsr^ can integrate on the extrachromosomal rDNA palindrome at nucleotide positions 18638 or 22168 (coordinates according to GenBank entry AY171066). These positions are nearly identical in DNA sequence and cannot be properly distinguished by PCR [19]. Inspecting the rDNA element at these positions revealed the presence of a consensus B box without any further similarity to tRNA genes. Subsequently, 21 additional B box motifs per 44 kb arm of the palindrome were determined (Fig 6). In the entire *D*. *discoideum* reference genome, 4,545 genomic positions can be detected when using the B box motif GTTCRANNC as the query, indicating that a B box-like motif can be expected every ~7,500 bp of genomic DNA. Thus, B box motifs appeared to be overrepresented on the rDNA element (22 in ~44 kb single arm of the palindrome), raising the question of whether all of these B boxes are functional and serve to assemble Pol III transcription complexes on the rDNA element. Our previous data indicated that a single B box is a poor target for TRE5-A integration, probably because it is inefficient in recruiting the Pol III transcription complex [19]. We hypothesized that functional A boxes must be present at B box loci to allow Pol III transcription complex formation as a requirement for TRE5-A integration. Efficient transcription of tRNA genes generally requires a critical distance between the A box and B box of 30–60 bp [35]. Therefore, we searched the 22 B box locations on the rDNA element for putative A boxes with this spacing. In fact, 14 A box-like sequences could be identified when using the most permissive sequence TNNNNNANNNG (Fig 6). Of these sequences, only six contained the more stringent TRRYNNARYNG motif characteristic of *D*. *discoideum* tRNA genes. These A/B box loci, 18638, 22168, 25439, 26202, 26963, and 27726, were apparently generated by a repeated duplication of larger segments of the rDNA palindrome as deduced from sequence conservation between these loci (S5 Fig). Although there is no sequence similarity of the discussed A/B box loci with tRNA genes despite the A box and B box motifs, these loci share another feature with tRNA genes: in 83% of *D*. *discoideum* tRNA genes, the first nucleotide of the mature tRNA is guanine and is located 7 bp upstream of the A box. Interestingly, a +1 G nucleotide is present at all six A/B box loci on the rDNA element (Fig 6). In the following, we will refer to the +1 G of an A/B box locus and calculate the distances of newly integrated TRE5-A elements from this position.

**Fig 7:**
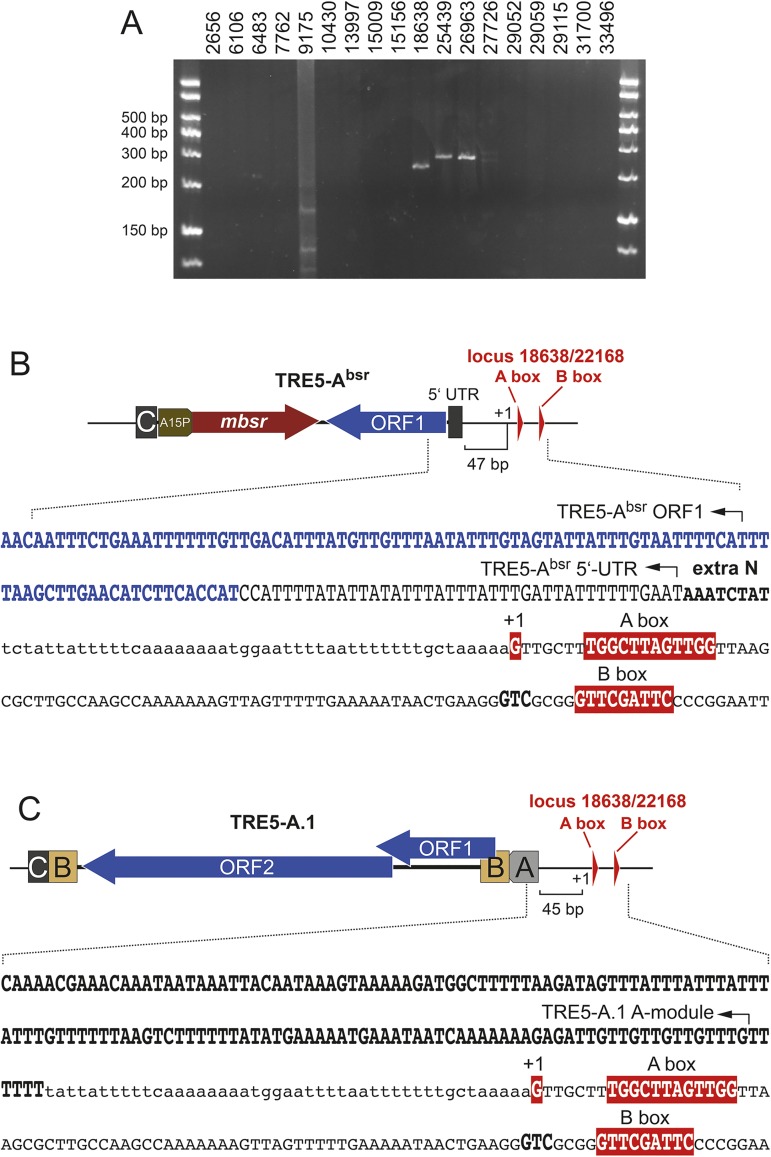
Determination of TRE5-A^bsr^ integrations sites on the rDNA palindrome. (**A**) The genomic DNA from a 15,000-clone pool of blasticidin-resistant cells was used for LAM-PCR-based enrichment of TRE5-A^bsr^ integrations. Exponential PCR amplification of the insertion junctions between TRE5-A^bsr^ ORF1 and any of the indicated B box positions was performed using locus-specific forward and ORF1-specific reverse primers. Note that a faint signal was reproducibly obtained at the 27726 locus that is barely visible in this reproduction (**B**) Example of an TRE5-A^bsr^ integration 47 bp upstream of the +1 G at position 18638/22168. The +1 G nucleotide, A box, and B box are indicated in red boxes. The integration junctions upstream of the +1 G are shown in lowercase letters. Note that the rDNA palindrome contains stretches of nearly identical sequences and thus B box positions 18638/22168 cannot be distinguished by PCR. (**C**) Integration of cellular TRE5-A.1 on the rDNA palindrome. The presence of a natural TRE5-A.1 element upstream of positions 18638/22168 on the rDNA palindrome was detected by nested PCR on genomic DNA of untransformed *D*. *discoideum* cells using primers specific for TRE5-A ORF1 and the 18638/22168 region. The +1 G nucleotide, A box, and B box are indicated in red boxes. The integration junctions upstream of the +1 G are shown in lowercase letters.

We used LAM-PCR products prepared from genomic DNA of the 100G culture to screen for the integration of TRE5-A^bsr^ at any of the 22 B box locations. To this end, we used primers for individual A/B box locations and a TRE5-A^bsr^-specific primer. PCR products indicative of TRE5-A^bsr^ insertions were obtained at 4 of 22 positions (Fig 7A). Because the sequences around positions 18638 and 25439 are repetitive and cannot be discriminated by PCR from the corresponding positions 22168 and 26202, six putative TRE5-A integration sites are located on each arm of the mirror-symmetric rDNA element (indicated by asterisks in Fig 6). Notably, these six integration sites coincide with the positions that best match the A/B box consensus: 18638, 22168, 25439, 26202, 26963, and 27726.

An example of a cloned and sequenced integration of TRE5-A^bsr^ at locus 18638/22168 is presented in Fig 7B. Using the tRNA gene-analogous +1 G as a measure for the 5' end of the targeted locus, the integration of TRE5-A^bsr^ occurred at a distance of 47 bp. Similar results were obtained from cloned integration junctions at loci 25439/26202, 26693, and 27726 (data not shown). Considering the data and structural similarity of the A/B box loci with typical tRNA genes, we concluded that TRE5-A^bsr^ elements recognized only positions with a functional A Box and approached these positions similar to conventional tRNA genes. Interestingly, TRE5-A^bsr^ integrations at positions 18638/22168 and 25439/26202 were also indicated in the Illumina sequences obtained from the 100G adapter primer library. Although only 242 pseudoreads mapped to these positions, evaluation of the integration junctions revealed an integration window of 37–50 bp relative to the +1 G at these positions (data not shown).

The annotation of the rDNA element did not present signs of previous TRE5-A integrations [27], perhaps due to bioinformatic consensus building, which discarded TRE5-A insertions in few of the ~100 palindrome copies present per cell. We therefore determined experimentally whether A/B box loci on the rDNA element are targeted by the natural TRE5-A population. We focused on position 18638/22168 and performed PCR on genomic DNA of untransformed *D*. *discoideum* AX2 cells. We used primers specific for the ORF1 sequence of the natural TRE5-A element and locus 18638/22168. After a conventional 30-cycle PCR, no product was detectable. However, nested PCR on this sample detected a small amount of specific PCR product (data not shown), which was cloned and sequenced. The obtained DNA sequence uncovered a TRE5-A.1 element integrated 45 bp upstream of the +1 G at locus 18638/22168 (Fig 7C). This result indicates that at least position 18638/22168 on the rDNA palindrome is a target for natural TRE5-A retrotransposition.

As mentioned above, our working hypothesis states that TRE5-A integrates at locations that assemble Pol III transcription complexes. According to this model, the A/B box positions on the rDNA palindrome represent previously unidentified active Pol III transcription units. To evaluate this possibility, we first performed northern blot analyses but could not detect any RNA transcripts derived from the TRE5-A^bsr^-targeted positions. Therefore, we conducted PCR with locus-specific synthesized cDNAs and detected transcripts at loci 18638/22168 (S6A Fig), 25439/26202, and 26963 (data not shown). To determine the lengths of the transcripts derived from these positions, we performed RT-PCR on circularized RNA (cRT-PCR) as outlined in S6B Fig. Products were obtained for all three loci. Two transcripts with different 5' ends were determined at loci 18638/22168; both map within the A box at positions 12 and 16 nt downstream of the +1 G (S6C Fig). We could not determine whether this result suggests 5'-processing, as in canonical tRNAs (mature tRNAs would start at the +1 G), or whether the isolated RNAs were prone to 5'-degradation either within the cells or upon RNA preparation. Two alternative 3' ends of 18638/22168 transcripts were determined after a T_5_ and T_6_ stretch, respectively, which are reminiscent of typical RNA polymerase III terminators. Overall, the 18638/22168 transcript length was approximately 230 nt. The 25439/26202 transcript started 22 nt downstream of the +1 G and ended at a T_5_ stretch, which would result in a 107-nt RNA (S6D Fig). The 5' end of the 26963 RNA was determined 1 nt upstream of the +1 G and terminated after 196 nt with no adjacent T-stretch present (S6D Fig).

In summary, the data indicate that TRE5-A can target locations on the rDNA palindrome because they represent functional A/B box motifs without a tRNA gene context and that these loci are probably occupied by transcription complexes and transcribed by Pol III.

## Discussion

### TRE5-A^bsr^ retrotransposition mimics the integration preference of natural TRE5-As

The determination of the authentic retrotransposition behavior of the TRE5-A element from genome data is hampered by frequent rearrangements during genome evolution. The comprehensive, experimental data obtained from two different in vivo integration assays in the present and previous studies [18, 19, 21] enable the further delineation of the integration preference of TRE5-A. Because TRE5-A^bsr^ contains the ORF1 protein that mediates target site detection [23] and borrows the ORF2 protein expressed by cellular TRE5-A.1 elements, it is plausible to assume that TRE5-A^bsr^ displays the same integration preference as the natural autonomous TRE5-A.1. This assumption is also supported by the observation that the non-autonomous TRE5-A.2 element displays the same integration behavior as TRE5-A.1 [18]. In this study, we determined a window of TRE5-A^bsr^ integration of 23–181 bp upstream of tRNA genes with 80.2% of TRE5-A^bsr^ integrations occurring within 47±3 bp upstream of the targeted tRNA genes (Fig 5). This observation was nicely consistent with the integration window of TRE5-As in the reference genome 37–211 bp upstream of tRNA genes (59.2% within the 47±3 bp window; n = 98) and experimentally determined de novo integrations of natural TRE5-A into the “TRE trap” (85.3% of integrations within the 47±3 bp window upstream of bait tRNA genes; n = 34) [18, 19]. Additional observations, including the formation of target site duplications (data not shown) and frequent insertion of non-templated extra nucleotides at the 5’ integration junction (Fig 3B), corroborated that TRE5-A^bsr^ mimics the natural integration of TRE5-A elements in the *D*. *discoideum* genome. In summary, we assume that position 47 upstream of tRNA genes represents the primary integration site of TRE5-A, which is immediately upstream the range that DNA-bound TFIIIB covers upstream of the transcription start site of tRNA genes [22].

In this study, we ruled out the possibility that TRE5-A expansion in the *D*. *discoideum* genome is restricted to a minority of tRNA genes because we determined that the TRE5-A^bsr^ element can reach almost every tRNA gene in the genome under experimental cell culture conditions. We speculate that TRE5-A expansion in the *D*. *discoideum* genome has not approached saturation because the resulting high copy number of retroelements might compromise genome stability. Because *D*. *discoideum* is haploid, there must be strong selection pressure to achieve steady-state TRE5-A amplification, which apparently leveled off at the reasonable average of 24% tRNA gene occupation observed in modern *D*. *discoideum* cells.

TRE5-A^bsr^ displays integration at canonical Pol III-transcribed genes, including the ribosomal 5S gene, and it further targets tRNA gene-like A/B box motifs on the extrachromosomal rDNA element, which may represent Pol III transcription units. We demonstrated in this study that the ribosomal 5S gene is a genuine target for integration by both the natural TRE5-A element and its derivative TRE5-A^bsr^. The window of TRE5-A^bsr^ integration relative to the 5' end of the 5S gene is 37–42 bp, which agrees well with the integration preference of natural TRE5-A elements 35–39 bp upstream of the 5S gene as determined in the “TRE trap assay” [19]. The somewhat shorter distance of TRE5-A integrations at the 5S gene compared with tRNA genes predicts that the positioning of TFIIIB is not identical on both gene types. In fact, TFIIIB occupies space on the *S*. *cerevisiae* 5S gene in the region from –40 to +5 [36].

An important issue in TRE5-A retrotransposition that could not be resolved in this study is whether TRE5-A may be directly mutagenic to protein-coding genes by performing “off-target” integration. A general drawback of LAM-PCR-based enrichment of integration junctions is the generation of the second DNA strand on LAM-PCR products, considering that the retrotransposon-upstream regions are unknown. Second-strand DNA synthesis using an adapter-linked hexamer primer obviously forced considerable amounts of artificial products during the subsequent amplification of TRE5-A^bsr^ integration junctions using the adapter primer on the distal ends of the LAM-PCR products. Whether this problem is related to the high A+T content of more that 80% in intergenic regions [12] remains unclear. Therefore it was impossible to discriminate whether mapping of Illumina sequence reads to open reading frames or intergenic regions without an A/B box context were generated by artificial fusion of genomic DNA to the retrotransposon during PCR or whether they indeed reflected off-target integration. However, we observed that it was easy to confirm predicted tRNA gene-directed integrations in samples of isolated blasticidin-resistant clones, whereas it was impossible to demonstrate insertions at predicted off-target sites.

Taking advantage of the orientation-specific integration of TRE5-A upstream of tRNA genes, the suitability of the LAM-PCR approach was demonstrated by the success of amplifying tDNA-TRE5-A^bsr^ integration junctions using tRNA gene-specific primers. Amplicon sequencing data from both the 20G and 100G tRNA library suggested hot spots of integration of TRE5-A^bsr^ at few tRNA genes. However, when comparing the two libraries there was almost no overlap of apparent “hot spots”. Interestingly, data from the 100G adpapter library reproduced this bias even though no tRNA gene primers were used in this experiments. Thus, we conclude that either a technical bias in PCR amplification of tRNA genes or expansion of certain clones in the population was resonsible for these observations.

### Do Pol III transcription complexes assemble on the extrachromosomal rDNA element?

In this work we detected integration of TRE5-A^bsr^ into the extrachromosomal rDNA element. Several lines of evidence suggest that TRE5-A^bsr^ targeted these loci as if they were tRNA genes. The binding of TFIIIC is a strict requirement for TRE5-A integration because a C56G mutation in the central GTTCRANNC motif of the B box abolishes TRE5-A integration [19]. Recent studies of “extra TFIIIC sites” (ETCs) have demonstrated that solo B boxes are enriched with bound TFIIIC but devoid of TFIIIB and Pol III [37–39]. In yeast, the retrotransposon Ty3 has been useful to indirectly confirm the absence of TFIIIB from ETCs [40]. The integration behavior of TRE5-A predicts that the B box loci on the extrachromosomal rDNA element do not represent ETC sites. Integration of TRE5-A upstream of a solo B box is possible but very inefficient [19], indicating that the A box plays an important role in positioning TFIIIC on a tRNA gene and thereby favors recruitment of TFIIIB as an interaction partner for TRE5-A. The position of the A box within a tRNA gene is fixed at position +8 relative to the first nucleotide (+1) of the mature tRNA. The precise integration of TRE5-A upstream of tRNA genes is likely mediated by the presence of the A box rather than by the position of the B box, which has a variable distance to the +1 nucleotide. The A box motif is more permissive than the B box consensus, which complicates the prediction of functional A boxes in Pol III genes. However, our data indicate that TRE5-A selected those B box positions on the extrachromosomal rDNA element that display the highest similarity to an A box while ignoring a majority of other B box positions. Although we do not have direct proof that Pol III transcription complexes assemble on the rDNA element, we speculate that targeting of TRE5-A upstream of A/B boxes at a tRNA gene-like distance predicts the presence of TFIIIC/TFIIIB complexes on the extrachromosomal rDNA palindrome. The observed formation of RNA transcripts covering TRE5-A target sites does not necessarily prove that these transcripts are Pol III products, but may indicate the presence of active transcription units other than the rRNA genes on the rDNA element.

Integration of TRE5-A^bsr^ into the extrachromosomal palindrome strongly predicts that the rDNA element is a target for integration by the endogenous TRE5-A population. We could detect integration of natural TRE5-A at rDNA loci targeted by TRE5-A^bsr^ in untransformed *D*. *discoideum* cells. However, the requirement of nested PCR to detect such integrations suggests that TRE5-A integrations have not become fixed in the rDNA element population, as also indicated by the lack of TRE5-A sequences determined during sequencing of the rDNA element [27]. Most eukaryotes organize their repetitive ribosomal RNA genes in head-to-tail clusters on one or several chromosomes. The puzzling observation that all copies of rDNA units in an isolate of a species are usually identical but may vary between different isolates has been explained by the hypothesis of concerted evolution, in which unequal homologous recombination and gene conversion homogenize the rDNA pool [41]. Amoebozoans appear to organize their rRNA genes in multiple copies of extrachromosomal elements in which two identical linear DNA arms are connected in a palindromic tail-to-tail arrangement. Therefore, it seems unlikely that unequal homologous recombination is involved in the homogenization of the extrachromosomal rDNA palindromes in *D*. *discoideum*. We believe that newly integrated mobile elements are rapidly removed from the rDNA element by gene conversion.

What roles may active Pol III complexes play in the biology of the extrachromosomal rDNA element? Notably, we also find A/B box-like arrangements on rDNA palindromes of other dictyostelids, such as *D*. *citrinum*, *D*. *purpureum*, *D*. *lacteum*, and *Polysphondylium pallidum* (data not shown). The conservation of A/B box arrangements over several hundreds of millions of years of evolution in a DNA element that harbors no transcription units other than rRNA genes is striking and strongly argues for conservation of a specific function. An attractive idea is that Pol III complexes may contribute to the replication of amoebozoan rDNA elements. Although the mechanism of replication of the palindromic rDNA element of *D*. *discoideum* is not known, it is clear from the data of Welker et al. [42] and analysis of the reference genome [12] that it is not produced from a chromosomal master copy as previously suggested by Sucgang et al. [27]. Bénard et al. have postulated distinct replication origins in the non-coding regions of the *Physarum* rDNA palindrome [43]. Combining this observation with data suggesting that tRNA genes can serve as replication fork pause sites [44, 45] may converge to an attractive model in which the A/B box loci on an extrachromosomal rDNA element regulate replication of the *D*. *discoideum* rDNA elements by transient Pol III complex formation.

## Conclusions

It is intriguing that integration at different distances upstream and downstream of tRNA genes has been invented at least twice by copia- and gypsy-like LTR retrotransposons in yeasts (e.g., Ty1 and Ty3) [46, 47] and at least six times in dictyostelid social amoebae by both LTR and non-LTR retrotransposons [48]. The combined knowledge of targeted integration by retrotransposons in fungi and amoebozoans provides strong support to the hypothesis that integration near tRNA genes (or Pol III genes in general) is an example of convergent evolution to solve the problem of colonizing high-density genomes without causing excessive gene mutagenesis. In this study we showed that retrotransposons tagged with the *mbsrI* gene can be followed to their natural integration sites, and that LAM-PCR-based transposon profiling is applicable to determine the integrome of mobile elements in the *D*. *discoideum* genome. The new data support our hypothesis that TRE5-A selects integration sites through interaction with the Pol III transcription complex. Integration near tRNA genes has been independently invented by non-LTR (TRE5, TRE3) and LTR (DGLT-A) retrotransposons during the evolution of *D*. *discoideum*. It will be interesting to perform retrotransposition profiling on the DGLT-A element, which has an integration preference upstream of tRNA genes that is different from TRE5-A but resembles that of the yeast Ty3 element [49]. The combination of *mbsrI*-tagged retrotransposons and integrome profiling presented in this study will foster research into the question whether tRNA genes do in fact present safe integration sites sites for mobile elements in compact genomes.

## Supporting information

S1 Fig Genomic distribution of tRNA genes in the *D*. *discoideum* reference genome. Information was extracted from „*D*. *discoideum* Non-coding sequences”as of 03-29-2016 (www.dictybase.org).
(TIF)

S2 Fig Enrichment of TRE5-A^bsr^ integrations by LAM-PCR. The genomic DNA from a pool of blasticidine-resistant cells carrying TRE5-A^bsr^ integrations was used as template for a linear PCR with a biotinylated primer that was designed to bind specifically to the codon-adapted ORF1 sequence of the cloned TRE5-A^bsr^ element. As negative control, a parallel LAM-PCR was performed in which the biotinylated ORF1 primer was missing. The linear, single-stranded LAM-PCR products were immobilized onto magnetic streptavidin beads, washed extensively and used as templates for exponential PCR to detect a single-copy gene (*gpdA*), the retrotransposon DIRS-1 (~200 copies per cell), the ORF1 sequence of endogenous TRE5-A elements, and TRE5-A^bsr^. As a positive control, exponential PCR was performed directly on genomic DNA.
(TIF)

S3 Fig Targeting of the ribosomal 5S gene by TRE5-A^bsr^. Example of an integration by TRE5-A^bsr^ upstream of the 5S gene on the rDNA element. Genomic DNA from ~15,000 blasticidin-resistant clones of the 20G culture was used for the LAM-PCR-based enrichment of TRE5-A^bsr^ integrations. The exponential PCR amplification of insertion junctions was performed between TRE5-A^bsr^ ORF1 and a primer that was specific for the 5S coding region. The 5S gene is shown in the red box. The sequence upstream of the 5S gene is shown in lowercase letters.
(TIF)

S4 Fig Targeting of the ribosomal 5S gene by cellular TRE5-A.1. The detection of a natural TRE5-A.1 element upstream of the 5S gene on the rDNA palindrome was performed on genomic DNA of untransformed *D*. *discoideum* cells using nested PCR with primers that were specific for TRE5-A ORF1 and the 5S gene coding region.The 5S gene is shown in the red box. The sequence upstream of the 5S gene is shown in lowercase letters.
(TIF)

S5 Fig Alignment of TRE5-A^bsr^-targeted B box loci on the rDNA palindrome. The alignment of DNA sequences from the indicated positions was performed with ClustalX, and the conserved nucleotide positions are highlighted using BoxShade. The positions of the +1G, A box and B box are indicated in red color.
(TIF)

S6 Fig Transcription of TRE5-A-targeted A/B box loci on the rDNA element. (**A**) RT-PCR result that was obtained from B box locus 18638/22168. cDNA was synthesized with a primer that was specific for that particular locus. Parallel reactions with (+) and without (–) the addition of RT in the reaction mixture were prepared. A PCR product that was produced on genomic DNA was used as the size marker (M). (**B**). Outline of the cRT-PCR protocol. The total RNA was prepared from the 15,000-clone pool of blasticidin-resistant cells. The RNA was treated for intramolecular ligation as detailed in the Materials and Methods section. Primer 1 was used for cDNA synthesis on circularized RNA. Then, PCR was performed on the cDNA using primers 2 and 3. The resulting DNA was purified and used as a template for nested PCR using primers 4 and 5. The products from this PCR were cloned and sequenced. Primers 1–5 for individual B box loci are listed in S1 Table. The sequencing results are shown for B-box locus 18638/22168 (**C**), and 25439/26202. The +1 Gs, A boxes and B boxes are indicated in red boxes. Oligothymidine stretches representing potential Pol III terminators are indicated as bold letters. The open arrowheads indicate the 5' ends of transcripts as deduced from cloned cRT-PCR products. The filled arrowheads indicate the 3' ends of transcripts.
(TIF)

S1 Table List of the primers used in this study. (PDF)

S2 Table Results from the mapping of TRE5-A^bsr^ integrations to tRNA gene loci. ^a^BF: floating contigs from chromosomes 4–6; ^b^2F: floating contigs of chromosome 2; abbreviations: Chr, chromosome; G, generations cell culture.
(PDF)

## References

[1] Deininger PL, Moran JV, Batzer MA, Kazazian HH. Mobile elements and mammalian genome evolution. Curr Opin Genet Dev. 2003; 13: 651–658. 14638329 10.1016/j.gde.2003.10.013

[2] Kazazian HH. Mobile elements: drivers of genome evolution. Science. 2004; 303: 1626–1632. doi: 10.1126/science.1089670 15016989 10.1126/science.1089670

[3] Cordaux R, Batzer MA. The impact of retrotransposons on human genome evolution. Nature Rev Genet. 2009; 10: 691–703. doi: 10.1038/nrg2640 19763152 10.1038/nrg2640 PMC2884099

[4] Levin HL, Moran JV. Dynamic interactions between transposable elements and their hosts. Nat Rev Genet. 2011; 12: 615–627. doi: 10.1038/nrg3030 21850042 10.1038/nrg3030 PMC3192332

[5] Mita P, Boeke JD. How retrotransposons shape genome regulation. Curr Opin Genet Dev. 2016; 37: 90–100. doi: 10.1016/j.gde.2016.01.001 26855260 10.1016/j.gde.2016.01.001 PMC4914423

[6] Xiong Y, Eickbush T. Origin and evolution of retroelements based upon their reverse transcriptase sequences. EMBO J. 1990; 9: 3353–3362. 1698615 10.1002/j.1460-2075.1990.tb07536.x PMC552073

[7] Eickbush TH. R2 and related site-specific non-long terminal repeat retrotransposons In: Craig NL, Craigie R, Gellert M, Lambowitz AM, editors. Mobile DNA II. Washington DC: ASM Press; 2002 p. 813–835.

[8] Moran JV, Gilbert N. Mammalian LINE-1 retrotransposons and related elements In: Craig NL, Craigie R, Gellert M, Lambowitz AM, editors. Mobile DNA II. Washington DC: ASM Press; 2002 p. 836–869.

[9] Feng QH, Schumann G, Boeke JD. Retrotransposon R1Bm endonuclease cleaves the target sequence. Proc Natl Acad Sci USA. 1998; 95(5): 2083–2088. 9482842 10.1073/pnas.95.5.2083 PMC19257

[10] Luan DD, Korman MH, Jakubczak JL, Eickbush TH. Reverse transcription of R2Bm RNA is primed by a nick at the chromosomal target site: a mechanism for non-LTR retrotransposition. Cell. 1993; 72: 595–605. 7679954 10.1016/0092-8674(93)90078-5

[11] Gilbert N, Lutz S, Morrish TA, Moran JV. Multiple fates of L1 retrotransposition intermediates in cultured human cells. Mol Cel Biol. 2005; 25: 7780–7795. 10.1128/MCB.25.17.7780-7795.2005 PMC1190285 16107723

[12] Eichinger L, Pachebat JA, Glöckner G, Rajandream M-A, Sucgang R, Berriman M, et al The genome of the social amoeba *Dictyostelium discoideum*. Nature. 2005; 435: 43–57. doi: 10.1038/nature03481 15875012 10.1038/nature03481 PMC1352341

[13] Glöckner G, Szafranski K, Winckler T, Dingermann T, Quail M, Cox E, et al The complex repeats of *Dictyostelium discoideum*. Genome Res. 2001; 11: 585–594. doi: 10.1101/gr.162201 11282973 10.1101/gr.162201 PMC311061

[14] Szafranski K, Glöckner G, Dingermann T, Dannat K, Noegel AA, Eichinger L, et al Non-LTR retrotransposons with unique integration preferences downstream of *Dictyostelium discoideum* transfer RNA genes. Mol Gen Genet. 1999; 262: 772–780. 10628860 10.1007/s004380051140

[15] Winckler T, Szafranski K, Glöckner G. Transfer RNA gene-targeted integration: an adaptation of retrotransposable elements to survive in the compact *Dictyostelium discoideum* genome. Cytogen Genome Res. 2005; 110: 288–298. 10.1159/000084961 16093681

[16] Kolosha VO, Martin SL. High affinity, non-sequence-specific RNA binding by the open reading frame 1 (ORF1) protein from long interspersed nuclear element 1 (LINE-1). J Biol Chem. 2003; 278: 8112–8117. doi: 10.1074/jbc.M210487200 12506113 10.1074/jbc.M210487200

[17] Martin SL, Bushman D, Wang F, Li PW-L, Walker A, Cummiskey J, et al A single amino acid substitution in ORF1 dramatically decreases L1 retrotransposition and provides insight into nucleic acid chaperone activity. Nucleic Acids Res. 2008; 18: 5845–5854. 10.1093/nar/gkn554 PMC2566875 18790804

[18] Beck P, Dingermann T, Winckler T. Transfer RNA gene-targeted retrotransposition of *Dictyostelium* TRE5-A into a chromosomal UMP synthase gene trap. J Mol Biol. 2002; 318: 273–285. doi: 10.1016/S0022-2836(02)00097-9 12051837 10.1016/S0022-2836(02)00097-9

[19] Siol O, Boutliliss M, Chung T, Glöckner G, Dingermann T, Winckler T. Role of RNA polymerase III transcription factors in the selection of integration sites by the *Dictyostelium* non-long terminal repeat retrotransposon TRE5-A. Mol Cell Biol. 2006; 26: 8242–8251. doi: 10.1128/MCB.01348-06 16982688 10.1128/MCB.01348-06 PMC1636787

[20] Schumann G, Zündorf I, Hofmann J, Marschalek R, Dingermann T. Internally located and oppositely oriented polymerase II promoters direct convergent transcription of a LINE-like retroelement, the *Dictyostelium* Repetitive Element, from *Dictyostelium discoideum*. Mol Cell Biol. 1994; 14: 3074–3084. 8164663 10.1128/mcb.14.5.3074-3084.1994 PMC358675

[21] Siol O, Spaller T, Schiefner J, Winckler T. Genetically tagged TRE5-A retrotransposons reveal high amplification rates and authentic target site preference in the *Dictyostelium discoideum* genome. Nucleic Acids Res. 2011; 39: 6608–6619. doi: 10.1093/nar/gkr261 21525131 10.1093/nar/gkr261 PMC3159450

[22] Geiduschek EP, Kassavetis GA. The RNA polymerase III transcription apparatus. J Mol Biol. 2001; 310: 1–26. doi: 10.1006/jmbi.2001.4732 11419933 10.1006/jmbi.2001.4732

[23] Chung T, Siol O, Dingermann T, Winckler T. Protein interactions involved in tRNA gene-specific integration of *Dictyostelium discoideum* non-long terminal repeat retrotransposon TRE5-A. Mol Cell Biol. 2007; 27: 8492–8501. doi: 10.1128/MCB.01173-07 17923679 10.1128/MCB.01173-07 PMC2169388

[24] Bentley DR, Balasubramanian S, Swerdlow HP, Smith GP, Milton J, Brown CG, et al Accurate whole human genome sequencing using reversible terminator chemistry. Nature. 2008; 456: 53–59. doi: 10.1038/nature07517 18987734 10.1038/nature07517 PMC2581791

[25] Ning Z, Cox AJ, Mullikin JC. SSAHA: a fast search method for large DNA databases. Genome Res. 2001; 11: 1725–1729. doi: 10.1101/gr.194201 11591649 10.1101/gr.194201 PMC311141

[26] Kent WJ. BLAT–the BLAST-like alignment tool. Genome Res. 2002; 12: 656–664. doi: 10.1101/gr.229202 11932250 10.1101/gr.229202 PMC187518

[27] Sucgang R, Chen GK, Liu W, Lindsay R, Lu J, Muzny D, et al Sequence and structure of the extrachromosomal palindrome encoding the ribosomal RNA genes in *Dictyostelium*. Nucleic Acids Res. 2003; 31: 2361–2368. 12711681 10.1093/nar/gkg348 PMC154234

[28] Robinson JT, Thorvaldsdóttir H, Winckler W, Guttman M, Lander ES, Getz G, et al Integrative genomics viewer. Nat Biotechnol. 2011; 29: 24–26. doi: 10.1038/nbt.1754 21221095 10.1038/nbt.1754 PMC3346182

[29] Boesler C, Kruse J, Söderbom F, Hammann C. Sequence and generation of mature ribosomal RNA transcripts in *Dictyostelium discoideum*. J Biol Chem. 2011; 286: 17693–17703. doi: 10.1074/jbc.M110.208306 21454536 10.1074/jbc.M110.208306 PMC3093845

[30] Silver J, Keerrikatte V. Novel use of polymerase chain reaction to amplify cellular DNA adjacent to an integrated provirus. J Virol. 190; 63: 1924–1928. 2704070 10.1128/jvi.63.5.1924-1928.1989 PMC250604

[31] Mueller PR, Wold B. In vivo footprinting of a muscle specific enhancer by ligation mediated PCR. Science. 1989; 246: 780–786. 2814500 10.1126/science.2814500

[32] Paruzynski A, Arens A, Gabriel A, Bartholomae C, Scholz S, Wang W, et al Genome-wide high-throughput integrome analyses by nrLAM-PCR and next-generation sequencing. Nat Protoc. 2010; 5: 1379–1395. doi: 10.1038/nprot.2010.87 20671722 10.1038/nprot.2010.87

[33] McKeown M, Firtel RA. Differential expression and 5' end mapping of actin genes in Dictyostelium. Cell. 1981; 24: 799–807. 6894715 10.1016/0092-8674(81)90105-7

[34] Chisholm RL, Gaudet P, Just EM, Pilcher KE, Fey P, Merchant SN, et al dictyBase, the model organism database for *Dictyostelium discoideum*. Nuc Acids Res. 2006; 34(Database issue): D423–D427. 10.1093/nar/gkj090 PMC1347453 16381903

[35] Baker RE, Camier S, Sentenac A, Hall BD. Gene size differentially affects the binding of yeast transcription factor t to two intragenic regions. Proc Natl Acad Sci USA. 1987; 87: 8768–8772. 10.1073/pnas.84.24.8768 PMC299631 2827154

[36] Costanzo G, Camier S, Carlucci P, Burderi L, Negri R. RNA polymerase III transcription complexes on chromosomal 5S rRNA genes in vivo: TFIIIB occupancy and promoter opening. Mol Cell Biol. 2001; 21(9): 3166–3178. doi: 10.1128/MCB.21.9.3166-3178.2001 11287621 10.1128/MCB.21.9.3166-3178.2001 PMC86947

[37] Moqtaderi Z, Struhl K. Genome-wide occupancy profile of the RNA polymerase III machinery in *Saccharomyces cerevisiae* reveals loci with incomplete transcription complexes. Mol Cell Biol. 2004; 24: 4118–4127. doi: 10.1128/MCB.24.10.4118-4127.2004 15121834 10.1128/MCB.24.10.4118-4127.2004 PMC400477

[38] Noma K, Cam HP, Maraia RJ, Grewal SI. A role for TFIIIC transcription factor complex in genome organization. Cell. 2006; 125: 859–872. doi: 10.1016/j.cell.2006.04.028 16751097 10.1016/j.cell.2006.04.028

[39] Moqtaderi Z, Wang J, Raha D, White RJ, Snyder M, Weng Z, et al Genomic binding profiles of functionally distinct RNA polymerase III transcription complexes in human cells. Nat Struct Biol. 2010; 17: 635–641. 10.1038/nsmb.1794 PMC3350333 20418883

[40] Qi X, Daily K, Nguyen K, Wang HX, Mayhew D, Rigor P, et al Retrotransposon profiling of RNA polymerase III initiation sites. Genome Res. 2012; 22: 681–692. doi: 10.1101/gr.131219.111 22287102 10.1101/gr.131219.111 PMC3317150

[41] Eickbush TH, Eickbush DG. Finely orchestrated movements: evolution of the ribosomal RNA genes. Genetics. 2007; 175: 477–485. doi: 10.1534/genetics.107.071399 17322354 10.1534/genetics.107.071399 PMC1800602

[42] Welker DL, Hirth KP, Williams KL. Inheritance of extrachromosomal ribosomal DNA during the asexual life cycle of Dictyostelium discoideum: Examination by use of DNA polymorphisms. Mol Cell Biol. 1985; 5: 273–280. 2983186 10.1128/mcb.5.2.273-280.1985 PMC366709

[43] Bénard M, Lagnel C, Pierron G. Site-specific initiation of DNA replication within the non-transcribed spacer of *Physarum* rDNA. Nucleic Acids Res. 1995; 23: 1447–1453. 7784195 10.1093/nar/23.9.1447 PMC306881

[44] Deshpande AM, Newlon CS. DNA replication fork pause sites dependent on transcription. Science. 1996; 272: 1030–1033. 8638128 10.1126/science.272.5264.1030

[45] Sekedat MD, Fenyö D, Rogers RS, Tackett AJ, Aitchison JD, Chait BT. GINS motion reveals replication fork progression is remarkably uniform throughout the yeast genome. Mol Syst Biol. 2010; 6: 353 doi: 10.1038/msb.2010.8 20212525 10.1038/msb.2010.8 PMC2858444

[46] Chalker DL, Sandmeyer SB. Ty3 integrates within the region of RNA polymerase III transcription initiation. Genes Dev. 1992; 6: 117–128. 1309715 10.1101/gad.6.1.117

[47] Devine SE, Boeke JD. Integration of the yeast retrotransposon Ty1 is targeted to regions upstream of genes transcribed by RNA polymerase III. Genes Dev. 1996; 10: 620–633. 8598291 10.1101/gad.10.5.620

[48] Spaller T, Kling E, Glöckner G, Hillmann F, Winckler T. Convergent evolution of tRNA gene targeting preferences in compact genomes. Mob DNA. 2016; 7: 17 doi: 10.1186/s13100-016-0073-9 27583033 10.1186/s13100-016-0073-9 PMC5006619

[49] Yieh L, Kassavetis GA, Geiduschek EP, Sandmeyer SB. The Brf and TATA-binding protein subunits of the RNA polymerase III transcription factor IIIB mediate position-specific Integration of the gypsy-like element, Ty3. J Biol Chem. 2000; 275: 29800–29807. doi: 10.1074/jbc.M003149200 10882723 10.1074/jbc.M003149200

