# Supplementary figures and images for: TRE5-A retrotransposition profiling reveals putative RNA polymerase III transcription complex binding sites on the Dictyostelium extrachromosomal rDNA element
Source: PLoS One. 2017 Apr 13;12(4):e0175729. doi: 10.1371/journal.pone.0175729 (PMC5391098; doi:10.1371/journal.pone.0175729)

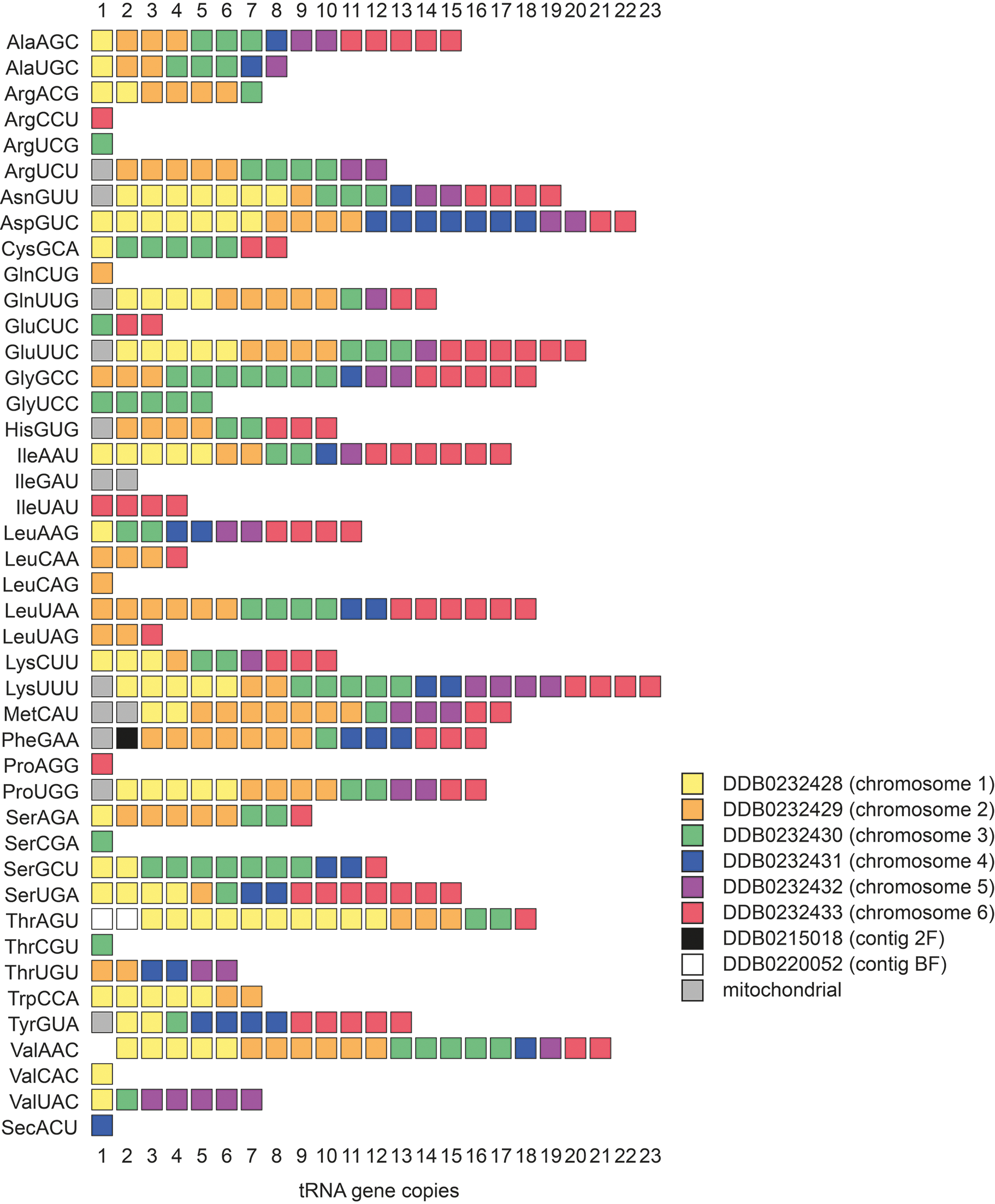

Supplement: S1 Fig — Information was extracted from „D. discoideum Non-coding sequences”as of 03-29-2016 (www.dictybase.org). (TIF) [file pone.0175729.s001.tif]

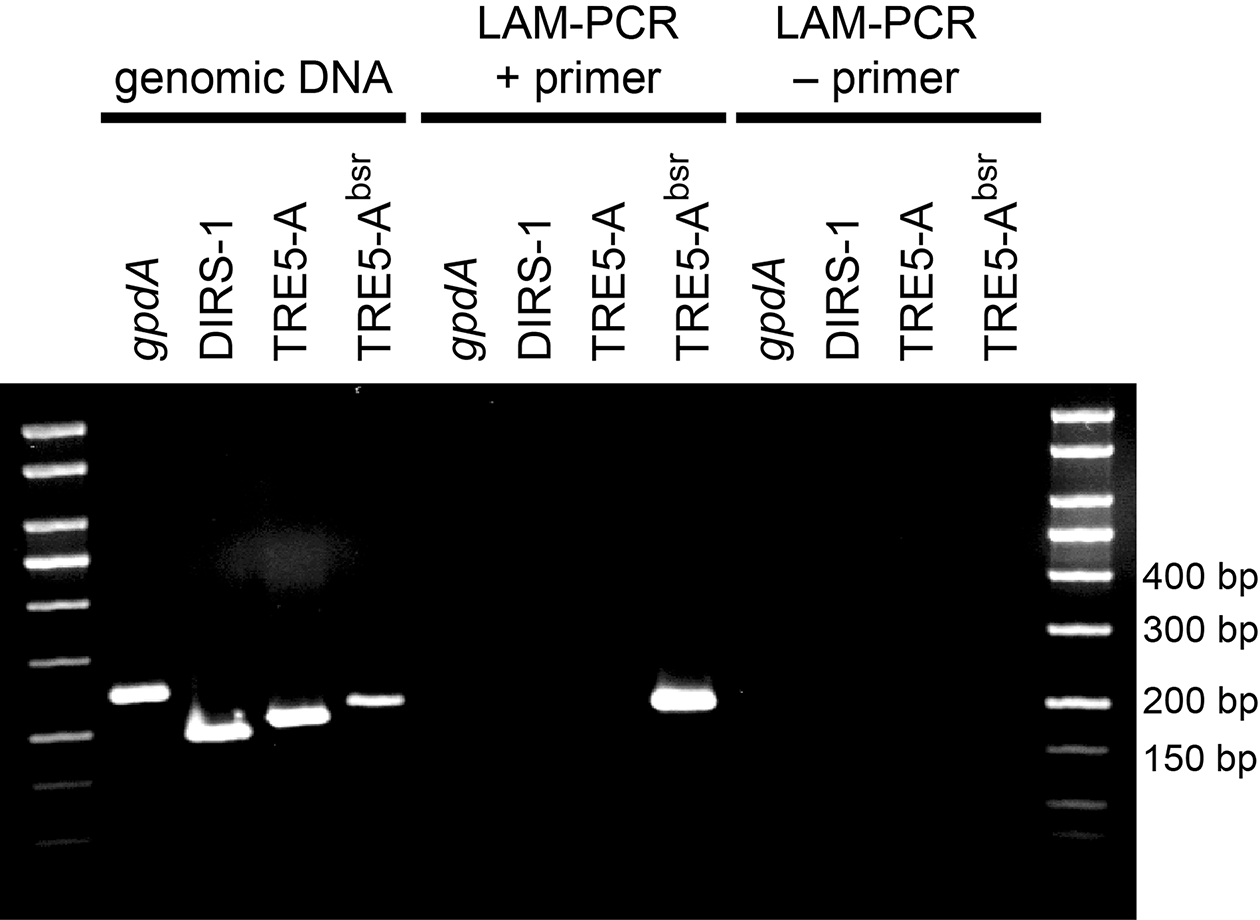

Supplement: S2 Fig — The genomic DNA from a pool of blasticidine-resistant cells carrying TRE5-Absr integrations was used as template for a linear PCR with a biotinylated primer that was designed to bind specifically to the codon-adapted ORF1 sequence of the cloned TRE5-Absr element. As negative control, a parallel LAM-PCR was performed in which the biotinylated ORF1 primer was missing. The linear, single-stranded LAM-PCR products were immobilized onto magnetic streptavidin beads, washed extensively and used as templates for exponential PCR to detect a single-copy gene (gpdA), the retrotransposon DIRS-1 (~200 copies per cell), the ORF1 sequence of endogenous TRE5-A elements, and TRE5-Absr. As a positive control, exponential PCR was performed directly on genomic DNA. (TIF) [file pone.0175729.s002.tif]

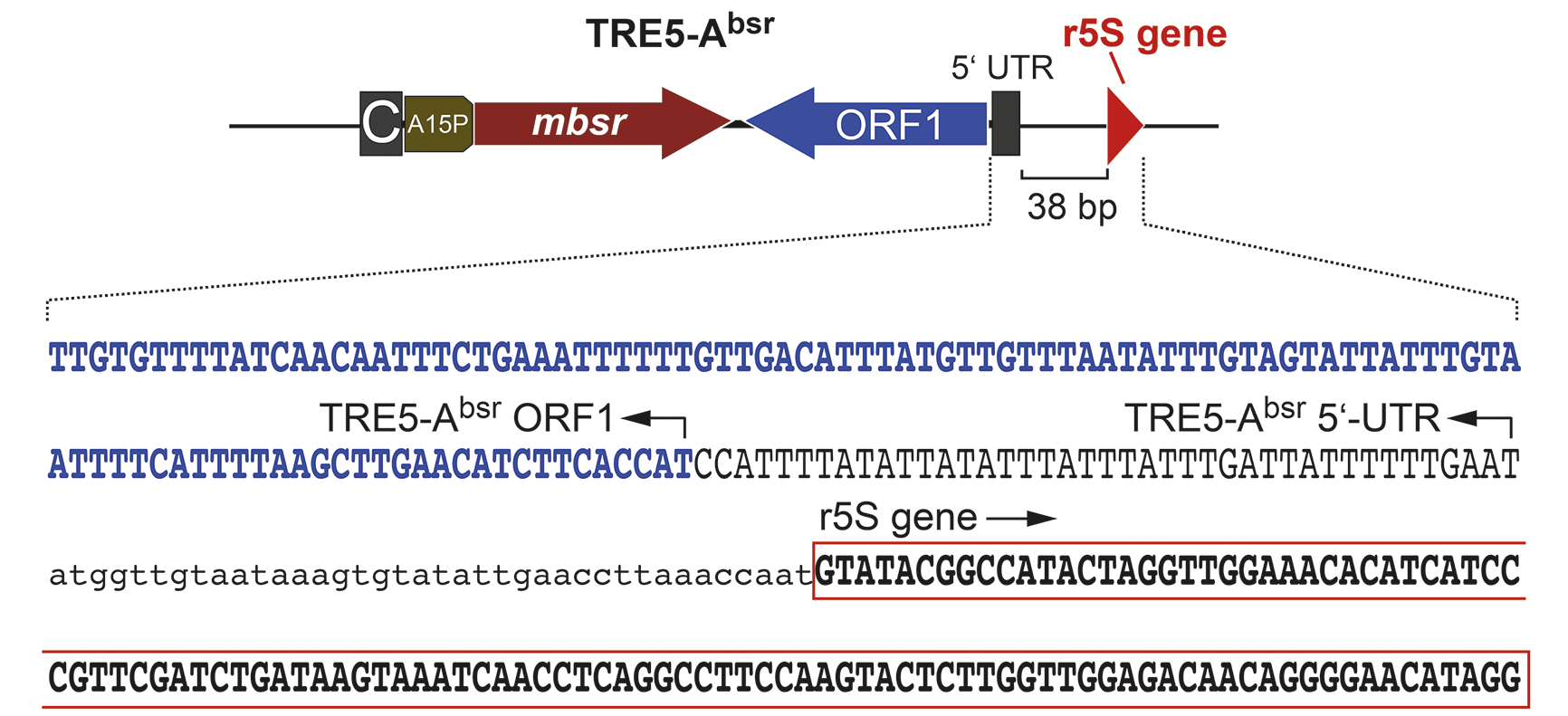

Supplement: S3 Fig — Example of an integration by TRE5-Absr upstream of the 5S gene on the rDNA element. Genomic DNA from ~15,000 blasticidin-resistant clones of the 20G culture was used for the LAM-PCR-based enrichment of TRE5-Absr integrations. The exponential PCR amplification of insertion junctions was performed between TRE5-Absr ORF1 and a primer that was specific for the 5S coding region. The 5S gene is shown in the red box. The sequence upstream of the 5S gene is shown in lowercase letters. (TIF) [file pone.0175729.s003.tif]

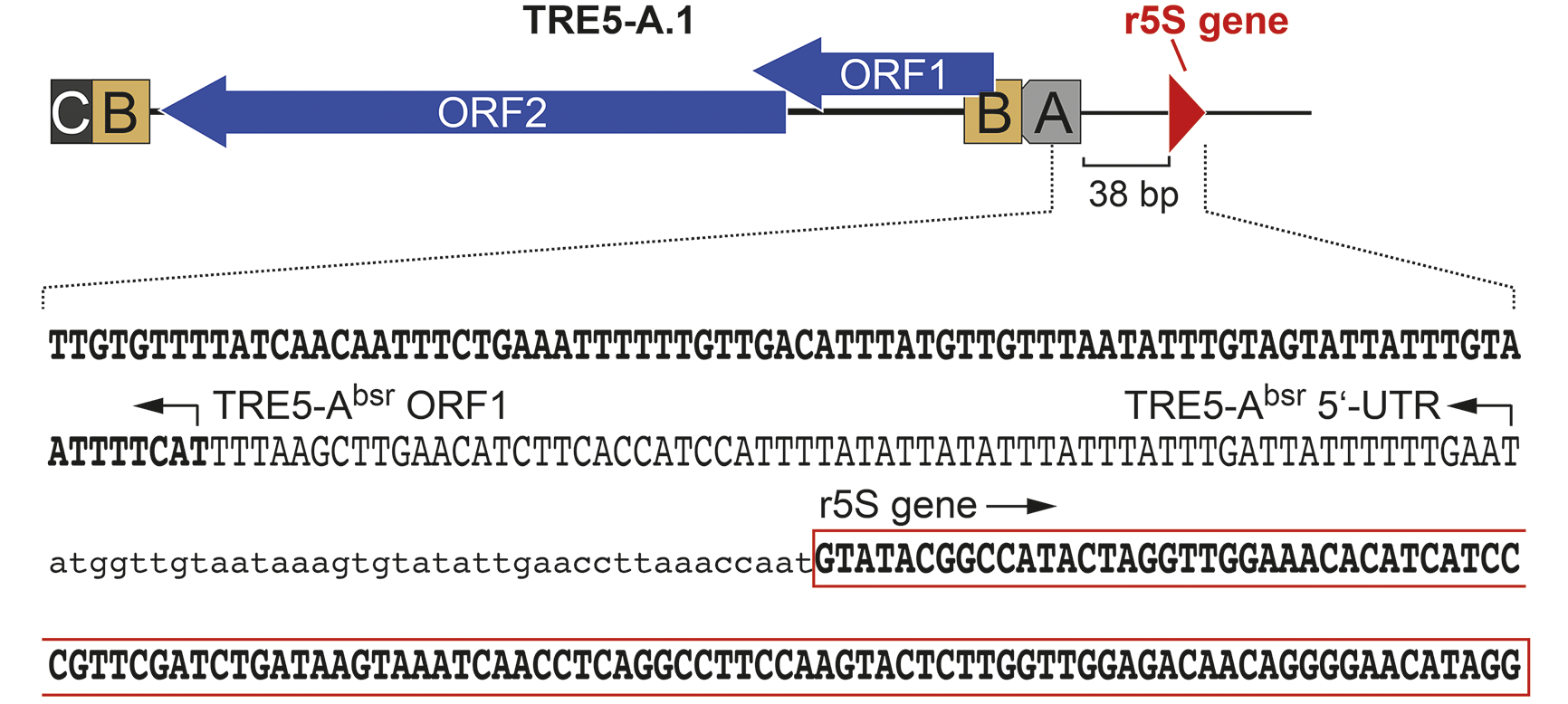

Supplement: S4 Fig — The detection of a natural TRE5-A.1 element upstream of the 5S gene on the rDNA palindrome was performed on genomic DNA of untransformed D. discoideum cells using nested PCR with primers that were specific for TRE5-A ORF1 and the 5S gene coding region.The 5S gene is shown in the red box. The sequence upstream of the 5S gene is shown in lowercase letters. (TIF) [file pone.0175729.s004.tif]

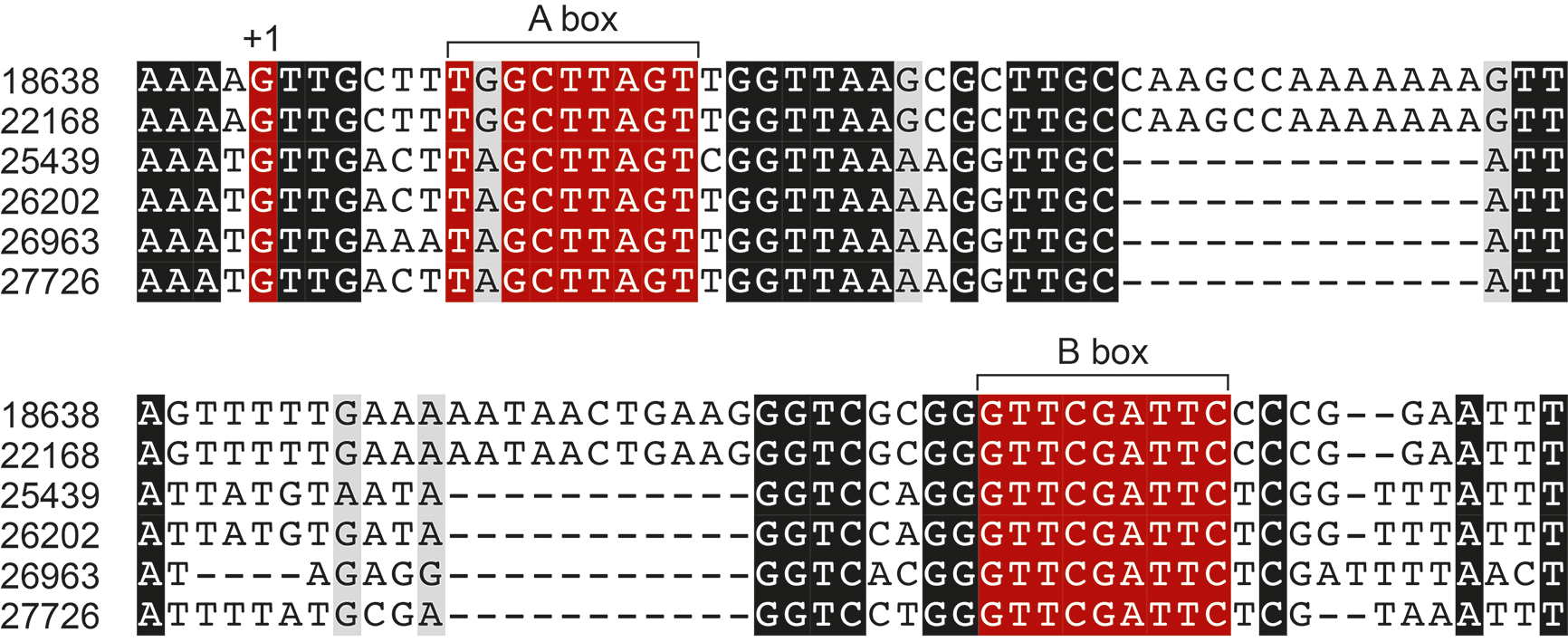

Supplement: S5 Fig — The alignment of DNA sequences from the indicated positions was performed with ClustalX, and the conserved nucleotide positions are highlighted using BoxShade. The positions of the +1G, A box and B box are indicated in red color. (TIF) [file pone.0175729.s005.tif]

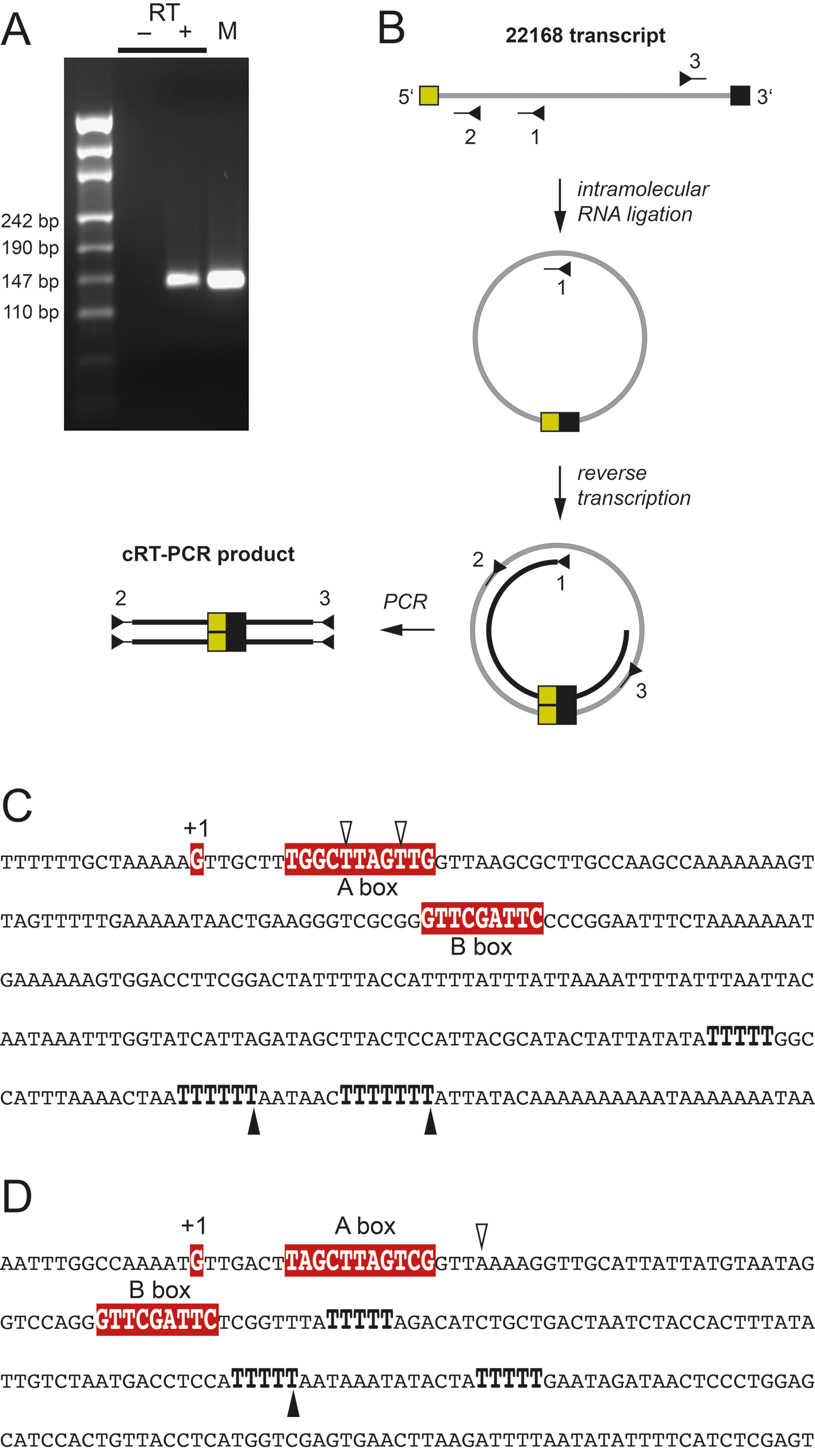

Supplement: S6 Fig — (A) RT-PCR result that was obtained from B box locus 18638/22168. cDNA was synthesized with a primer that was specific for that particular locus. Parallel reactions with (+) and without (–) the addition of RT in the reaction mixture were prepared. A PCR product that was produced on genomic DNA was used as the size marker (M). (B). Outline of the cRT-PCR protocol. The total RNA was prepared from the 15,000-clone pool of blasticidin-resistant cells. The RNA was treated for intramolecular ligation as detailed in the Materials and Methods section. Primer 1 was used for cDNA synthesis on circularized RNA. Then, PCR was performed on the cDNA using primers 2 and 3. The resulting DNA was purified and used as a template for nested PCR using primers 4 and 5. The products from this PCR were cloned and sequenced. Primers 1–5 for individual B box loci are listed in S1 Table. The sequencing results are shown for B-box locus 18638/22168 (C), and 25439/26202. The +1 Gs, A boxes and B boxes are indicated in red boxes. Oligothymidine stretches representing potential Pol III terminators are indicated as bold letters. The open arrowheads indicate the 5' ends of transcripts as deduced from cloned cRT-PCR products. The filled arrowheads indicate the 3' ends of transcripts. (TIF) [file pone.0175729.s006.tif]
