# Supplementary material for: TRE5-A retrotransposition profiling reveals putative RNA polymerase III transcription complex binding sites on the Dictyostelium extrachromosomal rDNA element
Source: PLoS One. 2017 Apr 13;12(4):e0175729. doi: 10.1371/journal.pone.0175729 (PMC5391098; doi:10.1371/journal.pone.0175729)
Supplement: S1 Table — (PDF) [file pone.0175729.s007.pdf]

Table S1: List of the primers used in this study.

Primer	Sequence (5'→3')
**LAM-PCR (TRE5-A ORF1-specific)**	
Rep-234_bio	Biotin-CAAAATAAATAACTGTTGAAATTG
Rep-235	CTTGAACATCTTCACCATCC
454B-6N	GCCTTGCCAGCCCGCTCAGNNNNNN
**PCR screening for TRE5-A^{bsr} integrations at tRNA gene families (LAM-PCR library)**	
AlaAGC	CCCTTTACCTCTCGCATGCTAAGC
AlaUGC	GATCCCTTTACCTTATGGAACTCTG
ArgACG	CTACCGGTTCGTAGCCGG
ArgCCU	GCATCTTATTCGTTAGGACCG
ArgUCU	CCTATTGATTCGAAGTCAATCGC
AsnGUU	GGACCTAGCGGTTAACAGCCGCTC
AspGUC	CCTTTCGCGTGACAGGCGAAAATC
CysGCA	GACCTAACGATTTGCAGTCGCTTG
GlnUUG	CAGATTACAGGATTCAAAGTCCTG
GluUUC	CGAGGTACCAGTGTGAAAGACTAG
GlyUCC	CGGTTCAACTGCTTGGAAGGCAGC
HisGUG	GGTCAACAGAGCCACAATCTGATG
IleAAU	CGATGCAGGGATCGAACCTGC
IleGAU	GCTTATCAAACGAATACTCTAACC
IleUAU	CTCGCGACCAAAGCATTGC
LeuUAA	GAACCGAATGGATGAGATCTTAAG
LeuUAG	CCTAAATCTATTGTCTTAACCAC
LysUUU	CCGACCACAAGGTTAAAAGCCTTG
MetCAU	GGACCTTAGGATTATGAGACC
PheGAA	CGACCTTAAGATCTTCAGTCTCAC
ProUGG	CCTATAATCTTTAGTTCCCAAAAC
SerAGA	CGAAGGGATCTGATTTCTAGTC
SerCGA	CTTTTATATGATTCGAGTCCAACTC
SerGCU	GAGAACGCCTTAGCAGGGC
SerUGA	GCGCCTCCTAGGAGATTTGATTTC
ThrAGU	GACCTCTTCCTTACTAGGGAAGCG
ThrCGU	GTATAATTTTATACGAGAGAAATGTCTTAC
ThrUGU	GACCCTCTGTTATCGCTTTAGCAC
TrpCCA	CTGGACCAACTGCTAACTTCTAAC
TyrGUA	CAACCGATTGATCTCAATTATTAC
ValAAC	ACGACCTAATCCGTGTTAAGGATT
ValCAC	GACCTAACCCGTGTGAAGGGC
ValUAC	GAGACCTGTTGCGTGTAAAGCAAC
**PCR screening for TRE5-A^{bsr} integrations at B-box loci on the rDNA palindrome**	
Pal-2656-rev	GAATTTAGAGATTAAAACTTTTTGGAG
Pal-6106-rev	CTTTAGTGCAGATCTTGATGGTAGTAG
Pal-6483-rev	GGGTATCATTTTAATTCACTTAATTGG
Pal-7762-rev	CACCTTCTACGACTCGAAATTCCAAAC
Pal-9175-rev	TGTGATGCCCTTAGATACCTTGGGCCG
Pal-10430-rev	GGTATTGATATTTATTGTCACTACTTC

Pal-13997-rev	CCCACTTTTTTGTACCCTTTGTAGGTC
Pal-15009-rev	TTCCTAAATGAGCAATTTGACATTCCC
Pal-15156-rev	AACAACTTTTTTAAGGTATAGTTACTC
Pal-18638-rev	AAATGGTAAAATAGTCCGAAGGTCCAC
Pal-25439-rev	GTGGATGCTCCAGGGAGTTATCTATTC
Pal-26963-rev	GTAGATGCCAATTGGAGTAATATATTC
Pal-27726-rev	TGAGGTAACAGTAGATTCTCCATTGAG
Pal-29052-rev	ATTAGGTCATAGGTTTCTGGATCGATC
Pal-29059-rev	CAACGGGGAATGGAAACGTCTTGAAAG
Pal-29115-rev	TCTAAGTTAGATTTTTACTAGTAATTC
Pal-31700-rev	TACCATTAGATAGCCCTATTTCTCTAG
Pal-33496-rev	GGAGTTGTCTATCCAACAAGGGTAATC
**PCR screening for TRE5-A^{bsr} integrations at the r5S gene on the rDNA palindrome**	
5S-rDNA-03	CCAACCAGAGTACTTGGAAGGC
**PCR screening for endogenous TRE5-A integrations at the r5S gene on the rDNA palindrome**	
5S-rDNA-04	GTATACAGCACCCTATGTTCCCCTG
Rep-190	CCATAGGTTGGGCTGAAACT
5S-rDNA-03	CCAACCAGAGTACTTGGAAGGC
Rep-109Q	GTTAGATTGTCTAGTTCAATGATAGTGTC
**PCR screening for endogenous TRE5-A integrations at B-box loci on the rDNA palindrome**	
Pal-06	GTAATGGAGTAAGCTATCTAATGATAC
Rep-190	CCATAGGTTGGGCTGAAACT
Pal-18638-rev	AAATGGTAAAATAGTCCGAAGGTCCAC
Rep-157Q	CTTCCGTTGTTAGTCTCTTTGTTTCGCC
**cRT-PCR Locus 18638**	
cRT-PCR_Pal-18638-01	AATGGTAAAATAGTCCGAAGGTCCAC
cRT-PCR_Pal-18638-02	CGAACCCGCGACCCTTCAG
cRT-PCR_Pal-18638-03	GTATCATTAGATAGCTTACTCCATTAC
cRT-PCR_Pal-18638-04	GTTGCTTTGGCTTAGTTGGTTAAGCG
cRT-PCR_Pal-18638-05	GCATACTATTATATATTTTT GGCC
**cRT-PCR Locus 25439**	
cRT-PCR_Pal-25439-01	CTATTACATAATAATGCAACC
cRT-PCR_Pal-25439-02	GAGAATCGAACCCTGGAC
cRT-PCR_Pal-25439-03	CTTTATATTGTCTAATGACCTCC
cRT-PCR_Pal-25439-04	GATGTCTAAAAATAAACCGAG
cRT-PCR_Pal-25439-05	TGCTGACTAATCTACCAC
**cRT-PCR Locus 26963**	
cRT-PCR_Pal-26963-01	GACCCCTCTATAATGCAACC
cRT-PCR_Pal-26963-02	AAAATCGAGAATCGAACCCG
cRT-PCR_Pal-26963-03	CAATTGGCATCTACCATTACC
cRT-PCR_Pal-26963-04	GGTGGATTAGTCATCATATGTC
cRT-PCR_Pal-26963-05	CTTTATGTGGTCTAATGACCTTC
**RT-PCR palindrome locus 18638/22168**	
Pal-RT_04	GTTGCTTTGGCTTAGTTGGTTAAGCG
Pal-RT_05	AATGGTAAAATAGTCCGAAGGTCCAC
