# Supplementary material for: TRE5-A retrotransposition profiling reveals putative RNA polymerase III transcription complex binding sites on the Dictyostelium extrachromosomal rDNA element
Source: PLoS One. 2017 Apr 13;12(4):e0175729. doi: 10.1371/journal.pone.0175729 (PMC5391098; doi:10.1371/journal.pone.0175729)
Supplement: S2 Table — aBF: floating contigs from chromosomes 4–6; b2F: floating contigs of chromosome 2; abbreviations: Chr, chromosome; G, generations cell culture. (PDF) [file pone.0175729.s008.pdf]

Table S2: Results from the mapping of TRE5-A^{bsr} integrations to tRNA gene loci.

[a]BF: floating contigs from chromosomes 4-6; [b]2F: floating contigs of chromosome 2; abbreviations: Chr, chromosome; G, generations cell culture.

tRNA gene	Chr	Positions of tRNA genes		Mapped contigs (counts)		
				20G tDNA primers	100G tDNA primers	100G adapter primer
ThrAGU-1	BF[a]	33501	33572	3	132	76
ThrAGU-2	BF[a]	33977	34048	10	336	122
LysUUU-2	1	448541	448611	6	556	272
ThrAGU-3	1	735884	735955	0	401	28
AsnGUU-8	1	750033	750105	1	23	6
SerUGA-1	1	818727	818808	4	16	0
CysGCA-1	1	818921	818992	0	0	3
SerUGA-4	1	822333	822414	0	420	29
ProUGG-2	1	835755	835825	18	3	0
ProUGG-3	1	843216	843286	0	0	9
SerUGA-2	1	946092	946173	3	3347	199
TrpCCA-5	1	1508042	1508128	0	0	3
TrpCCA-4	1	1511351	1511437	12	672	20
ProUGG-6	1	1615509	1615579	18	0	266
ProUGG-5	1	1618454	1618524	20506	0	13
AsnGUU-2	1	1632774	1632846	0	0	4
TyrGUA-2	1	1686235	1686317	1	0	0
ThrAGU-4	1	1688222	1688293	1	4	0
LysCUU-1	1	1759767	1759839	3	0	162
ArgACG-2	1	1760348	1760421	43221	3528	396
ThrAGU-5	1	1863435	1863506	109	143	3
AsnGUU-3	1	1956750	1956822	0	118	12
AsnGUU-4	1	1970455	1970527	0	0	0
ThrAGU-6	1	1995560	1995631	36	14	0
ThrAGU-7	1	1997247	1997318	2842	0	0
ArgACG-1	1	2023312	2023385	4	203	26
ValAAC-6	1	2064611	2064684	0	129	5
ValUAC-1	1	2073265	2073338	0	0	0
AspGUC-7	1	2099322	2099393	0	41	27
ValAAC-5	1	2099470	2099543	0	1725	70
MetCAU-4	1	2164854	2164925	0	1	1990
MetCAU-3	1	2166447	2166518	0	0	98
AspGUC-6	1	2265532	2265603	57	162	29
AspGUC-1	1	2278096	2278167	0	54	9
GluUUC-2	1	2366931	2367002	4126	25	0
GluUUC-6	1	2368298	2368369	3504	81	0
GlnUUG-5	1	2370125	2370197	2	16	244
GlnUUG-2	1	2371018	2371090	0	5	30
AsnGUU-7	1	2390365	2390437	0	45	7
LysUUU-3	1	2398344	2398416	339	289	151
ThrAGU-8	1	2404737	2404808	39	8	0
TrpCCA-3	1	2411874	2411960	1097	727	22
SerGCU-1	1	2414216	2414296	5	86	5
SerGCU-2	1	2424793	2424873	239	6166	217
LysUUU-4	1	2503577	2503649	31	84	25

SerAGA-1	1	2558667	2558748	3	1596	91
LysCUU-3	1	2560506	2560578	0	0	5
GluUUC-3	1	2687279	2687350	14	947	127
LysCUU-2	1	2697090	2697162	33	0	799
GluUUC-5	1	2698672	2698743	0	8	0
TrpCCA-1	1	2817548	2817634	91	286	17
GlnUUG-3	1	2850044	2850116	0	0	3
GlnUUG-4	1	2855252	2855324	59	4162	112
LysUUU-5	1	2877753	2877825	17	3140	1663
TrpCCA-2	1	2913067	2913153	20	521	55
SerUGA-3	1	2967080	2967161	0	251	206
GluUUC-4	1	3008412	3008483	51	4031	144
ThrAGU-9	1	3222819	3222890	9	394	24
IleAAU-5	1	3256805	3256877	7900	314	19
ProUGG-4	1	3314085	3314155	1	0	0
AsnGUU-6	1	3431040	3431112	0	218	70
AlaAGC-1	1	3431190	3431262	345	79	17
AsnGUU-5	1	3579771	3579843	32	23038	28064
ThrAGU-12	1	3644185	3644256	4092	81177	5123
AspGUC-2	1	3865846	3865917	9	45	2
ValCAC-1	1	3928023	3928095	39958	9925	170
TyrGUA-3	1	4009415	4009497	0	5	63
IleAAU-1	1	4125226	4125298	0	75	43
LysUUU-6	1	4158870	4158942	43	383	142
IleAAU-4	1	4163571	4163643	90	245	126
AlaUGC-1	1	4185581	4185676	6	224	2
AspGUC-3	1	4201373	4201444	1	100	30
ThrAGU-10	1	4214694	4214765	0	28	4
AspGUC-5	1	4249798	4249869	11	4126	494
AspGUC-4	1	4250554	4250625	3	4150	305
ValAAC-4	1	4298314	4298387	0	123	7
ValAAC-3	1	4301392	4301465	1	690	35
ValAAC-2	1	4338908	4338981	0	0	0
IleAAU-3	1	4551714	4551786	0	0	0
IleAAU-2	1	4562415	4562487	0	6	0
ThrAGU-11	1	4709052	4709123	0	37	0
PheGAA-2	2F[b]	122350	122423	0	0	0
ValAAC-12	2	347022	347095	20	240	3
ValAAC-11	2	386413	386486	0	0	0
ArgUCU-2	2	618267	618339	0	0	6
GlyGCC-3	2	996180	996250	0	12	13
ProUGG-10	2	1365573	1365643	0	2	250
PheGAA-9	2	1465351	1465424	0	55	11
IleAAU-6	2	1492518	1492590	5	31	0
GlnUUG-6	2	1825599	1825671	1	6	83
PheGAA-3	2	2492015	2492088	0	0	0
GluUUC-7	2	2498149	2498220	0	2	0
LeuUAG-2	2	2506873	2506951	1	6	3
MetCAU-11	2	2513207	2513278	0	0	4
MetCAU-5	2	2516614	2516685	0	0	1
LeuUAA-1	2	2661584	2661666	3	0	0
ThrAGU-13	2	2662753	2662824	0	0	0
ThrAGU-15	2	3368962	3369033	0	3	0
LeuUAA-6	2	3370120	3370202	3	12	7

MetCAU-10	2	3515101	3515172	0	3	2
MetCAU-6	2	3518508	3518579	0	0	13
LeuUAG-1	2	3524835	3524913	0	3	3
GluUUC-10	2	3533566	3533637	0	3	0
PheGAA-8	2	3539698	3539771	0	0	0
GlnUUG-10	2	3853359	3853431	0	1	4
LeuUAA-2	2	4103357	4103439	41	855	226
GlyGCC-2	2	4109270	4109340	0	0	7
GlyGCC-1	2	4356024	4356094	0	0	30
SerAGA-2	2	4399881	4399962	342	813	74
MetCAU-9	2	4972878	4972950	1	3820	481
PheGAA-4	2	4981255	4981328	0	15	6
LysUUU-8	2	4989791	4989863	0	33	8
TrpCCA-6	2	4995095	4995181	681	583	1257
GlnCUG-1	2	5171836	5171924	0	0	13
AspGUC-11	2	5197716	5197787	0	9	12
ThrUGU-1	2	5288579	5288668	0	0	121
HisGUG-5	2	5290018	5290088	25	11	0
MetCAU-8	2	5314110	5314182	0	3204	414
LysCUU-4	2	5400772	5400844	0	0	4
MetCAU-7	2	5416778	5416850	919	6066	573
GluUUC-9	2	5452266	5452337	18	9305	201
GluUUC-8	2	5454647	5454718	24	1001	287
LeuCAA-3	2	5489684	5489764	1	0	92
LeuCAA-1	2	5492551	5492631	1	33	1726
TrpCCA-7	2	5820963	5821049	5	2726	1406
GlnUUG-9	2	5907958	5908030	0	34	348
AsnGUU-9	2	5934172	5934244	1	131	55
HisGUG-4	2	5947196	5947266	0	1	1
ValAAC-7	2	6127229	6127302	51	308	40
AspGUC-8	2	6132328	6132399	10	30	13
ValAAC-10	2	6162014	6162087	0	23	1
ArgUCU-3	2	6187314	6187386	13	0	65
ArgUCU-6	2	6227029	6227101	0	0	46
GlnUUG-8	2	6364519	6364591	1	18	108
SerAGA-3	2	6379305	6379386	0	1024	22
ArgACG-3	2	6386977	6387050	12	1021	178
SerUGA-5	2	6473009	6473086	0	380	272
LeuCAA-2	2	6482159	6482239	18	4	143
SerAGA-4	2	6571351	6571432	0	7202	284
ArgUCU-4	2	6640387	6640459	0	0	3
LeuUAA-5	2	6646491	6646573	0	193	44
ArgUCU-5	2	6647152	6647224	1	1	62
ThrUGU-2	2	6680526	6680615	143	1783	927
GlnUUG-7	2	6705104	6705176	1	0	27
ArgACG-6	2	6724130	6724203	18	35	14
PheGAA-5	2	6766972	6767045	0	13	0
ProUGG-9	2	6771543	6771613	0	0	23
PheGAA-7	2	6771686	6771759	0	63	14
LysUUU-7	2	6863248	6863320	0	1332	350
LeuUAA-4	2	6948162	6948244	0	78	20
ProUGG-7	2	7004296	7004366	230	0	250
AlaAGC-2	2	7122699	7122771	4	1371	103
LeuCAG-1	2	7144841	7144924	0	0	1

PheGAA-6	2	7187981	7188054	1	2622	712
SerAGA-6	2	7237204	7237285	6	401	14
AlaUGC-2	2	7430263	7430358	0	22	0
AlaUGC-3	2	7434539	7434634	0	0	0
ValAAC-8	2	7435671	7435744	51	22	2
ArgACG-4	2	7473728	7473801	0	221	62
ProUGG-8	2	7524451	7524521	0	0	18
IleAAU-7	2	7651928	7652000	1	56	14
AspGUC-10	2	7675988	7676059	3	49	8
AspGUC-9	2	7715120	7715191	0	1202	398
LeuAAG-1	2	7738676	7738757	0	0	18
ArgACG-5	2	7837550	7837623	1	713	92
ValAAC-9	2	7845634	7845707	0	0	0
LeuUAA-3	2	7871755	7871837	4	22599	8957
SerAGA-5	2	7878701	7878782	12	7298	261
ThrAGU-14	2	7936345	7936416	0	19	3
HisGUG-3	2	8084436	8084506	0	282	47
AlaAGC-3	2	8141487	8141559	29	852	36
HisGUG-2	2	8290826	8290896	0	3	9
AlaAGC-4	2	8364904	8364976	8	6337	406
TyrGUA-4	3	506383	506465	0	6	143
HisGUG-6	3	549066	549136	19	17	2
AsnGUU-10	3	1045240	1045312	0	76	44
LysUUU-13	3	1112773	1112845	17	36	2
LysUUU-12	3	1431187	1431259	45	617	268
CysGCA-6	3	1574082	1574153	5	1628	123
CysGCA-5	3	1671338	1671409	0	56	9
ArgUCG-1	3	1773594	1773667	0	3072	30
ValUAC-2	3	1807996	1808069	33	1923	28
GlyGCC-10	3	2103501	2103571	0	0	193
GluUUC-13	3	2156654	2156725	0	1110	41
CysGCA-2	3	2330677	2330748	0	30220	4577
AsnGUU-11	3	2443914	2443986	0	1195	717
CysGCA-3	3	2450297	2450368	7	281	18
SerAGA-8	3	2539790	2539871	6	5196	206
His-GUG-7	3	2623274	2623344	0	0	0
CysGCA-4	3	2632482	2632553	4	4446	177
GluUUC-12	3	2675536	2675607	4289	228	29
IleAAU-9	3	2700535	2700607	4	328	79
ValAAC-13	3	2745400	2745473	0	0	0
ArgACG-7	3	2876124	2876197	11	64	6
GlyGCC-4	3	2940647	2940717	4	0	1
AsnGUU-12	3	3060646	3060718	0	2702	487
ArgUCU-10	3	3102884	3102956	0	0	0
SerAGA-7	3	3143611	3143692	7	321	9
GlnUUG-11	3	3366146	3366218	125	437	6226
LysUUU-11	3	3574246	3574318	0	1	0
GlyGCC-5	3	3875955	3876025	0	0	5
GlyGCC-6	3	3876679	3876749	34	0	9
GluUUC-11	3	3889342	3889413	0	19	1
AlaAGC-7	3	3993886	3993958	8	2690	88
AlaAGC-5	3	4014778	4014850	5	454	57
AlaAGC-6	3	4067961	4068033	224	32	4
AlaUGC-6	3	4090199	4090294	5993	549	47

AlaUGC-4	3	4194528	4194623	37	2216	198
GlyUCC-1	3	4240788	4240858	7	571	2
GlyUCC-5	3	4247160	4247230	2994	143	3
LysCUU-5	3	4266691	4266763	104	0	25
LysUUU-10	3	4268093	4268165	5699	158	111
LysUUU-9	3	4270315	4270387	8	649	189
AlaUGC-5	3	4297271	4297366	0	3359	200
PheGAA-10	3	4353450	4353523	0	3	1
GlyGCC-9	3	4400356	4400426	1	0	1
GlyGCC-7	3	4413344	4413414	0	0	12
LeuUAA-7	3	4458032	4458114	6	1466	353
LeuUAA-10	3	4511889	4511971	0	134	126
LeuAAG-2	3	4523794	4523875	1	0	88
GlyGCC-8	3	4565971	4566041	0	0	9
ArgUCU-9	3	4567934	4568006	869	0	8
LeuUAA-8	3	4606291	4606373	0	88	48
LeuUAA-9	3	4613433	4613515	0	39	41
MetCAU-12	3	4660155	4660226	0	2	169
ThrCGU-1	3	5070744	5070829	27	15	45
ArgUCU-8	3	5126146	5126218	1	0	26
GluCUC-1	3	5321663	5321734	1	1	0
GlyUCC-4	3	5325920	5325990	9518	92	3
SerGCU-9	3	5333581	5333661	42	15002	1675
SerGCU-8	3	5335122	5335202	16	1987	57
SerGCU-7	3	5339366	5339446	1498	16237	372
ProUGG-11	3	5518229	5518299	0	0	1
ProUGG-12	3	5519054	5519124	0	0	0
LysCUU-6	3	5652919	5652991	0	0	51
ArgUCU-7	3	5654004	5654076	1	0	807
SerCGA-1	3	5679957	5680052	4250	11767	943
GlyUCC-3	3	5706517	5706587	760	16826	536
GlyUCC-2	3	5706954	5707024	802	9049	212
SerUGA-6	3	5751386	5751467	0	391	11
SerGCU-6	3	5781580	5781660	29	8636	373
ValAAC-14	3	5868008	5868081	107	273	10
ValAAC-15	3	5870128	5870201	14	461	10
SerGCU-3	3	5898457	5898537	34	3093	61
SerGCU-4	3	5902871	5902951	22	433	28
SerGCU-5	3	5906903	5906983	73	534	30
ThrAGU-16	3	5918532	5918603	0	88	9
LeuAAG-3	3	5937248	5937329	0	0	0
ValAAC-17	3	5940507	5940580	1	166	4
ThrAGU-17	3	5981778	5981849	0	652	75
IleAAU-8	3	6072152	6072224	0	160	41
ValAAC-16	3	6078780	6078853	0	0	0
SerUGA-8	4	1589109	1589190	4	856	32
LysUUU-15	4	1802373	1802445	10	69	41
IleAAU-10	4	1879566	1879638	1	216	54
SerGCU-11	4	2220197	2220277	240	5132	259
SerGCU-10	4	2227631	2227711	14	106	19
PheGAA-11	4	2563537	2563610	5	1280	403
AspGUC-12	4	2841024	2841095	0	76	22
AspGUC-18	4	2845703	2845774	8	35	0
AspGUC-17	4	3029253	3029324	0	19	38

AspGUC-16	4	3057091	3057162	0	6731	1509
AspGUC-13	4	3073029	3073100	0	133	23
GlyGCC-11	4	3320060	3320130	0	0	0
AsnGUU-13	4	3320206	3320278	0	5	1
TyrGUA-5	4	3366227	3366309	0	4	1135
TyrGUA-6	4	3658459	3658541	0	0	15
ThrUGU-3	4	3710357	3710446	0	0	0
ThrUGU-4	4	3717164	3717253	0	5	2
AspGUC-15	4	3919822	3919893	11	2313	370
AspGUC-14	4	3942404	3942475	7	337	36
SerUGA-7	4	3945723	3945804	1	1400	55
AlaAGC-8	4	3947721	3947793	241	6163	526
LysUUU-14	4	3970863	3970935	14	422	66
PheGAA-12	4	4598294	4598367	2	390	184
AlaUGC-7	4	4682789	4682884	32	1603	160
TyrGUA-7	4	4844157	4844240	2	0	64
PheGAA-13	4	4999193	4999266	0	171	32
ValAAC-18	4	5047177	5047250	0	0	0
LeuUAA-11	4	5168273	5168355	102	241	105
LeuUAA-12	4	5226859	5226941	12	13266	6957
TyrGUA-8	4	5312947	5313029	7	14	243
LeuAAG-4	4	5435309	5435390	0	0	229
LeuAAG-5	4	5435936	5436017	0	0	0
LysUUU-16	5	40886	40958	55	116	167
AlaAGC-10	5	254727	254799	11	84	2
MetCAU-15	5	268971	269043	1	102	14
IleAAU-11	5	278611	278683	12	47	20
GlyGCC-12	5	284121	284191	0	0	0
GlyGCC-13	5	409584	409654	0	0	0
LeuAAG-7	5	432078	432159	12	0	945
ThrUGU-6	5	712716	712805	546	11	1
AsnGUU-15	5	1592074	1592146	0	39	52
GlnUUG-12	5	1989890	1989962	0	13	60
AlaAGC-9	5	2265374	2265446	0	142	5
AsnGUU-14	5	2321011	2321083	0	49	16
LeuAAG-6	5	2550132	2550213	4	0	120
ArgUCU-12	5	2593624	2593696	5	0	196
LysUUU-17	5	2727390	2727462	3	52	83
AspGUC-20	5	2728188	2728259	10	2376	429
LysUUU-18	5	2768640	2768712	9	108	26
ProUGG-13	5	2956680	2956750	2	0	3
ProUGG-14	5	2970447	2970517	7	0	132
ValUAC-3	5	3061365	3061438	5	1962	464
LysUUU-19	5	3113136	3113208	17	698	56
ValAAC-19	5	3208187	3208260	82	339	4
GluUUC-14	5	3245733	3245804	3	593	109
MetCAU-13	5	3259687	3259759	0	7520	1158
ThrUGU-5	5	3336512	3336601	137	246	147
ValUAC-4	5	3658887	3658960	0	7	8
ValUAC-5	5	3660287	3660360	2	105	34
ArgUCU-11	5	3693576	3693648	0	24	46
ValUAC-6	5	3761819	3761892	288	8	6
MetCAU-14	5	3845621	3845693	0	6	17
AlaUGC-8	5	3987062	3987157	2206	7	8

AspGUC-19	5	4005478	4005549	0	205	29
LysCUU-7	5	4947710	4947782	4	0	45
LeuCAA-4	6	310825	310905	0	0	0
GluUUC-15	6	323423	323494	23	650	128
ValAAC-20	6	586137	586210	6	2657	47
LeuUAG-3	6	604764	604843	0	0	0
GlyGCC-14	6	859158	859228	53	0	0
PheGAA-16	6	898266	898339	0	147	1193
IleAAU-17	6	1013913	1013985	0	12	11
LeuUAA-18	6	1044809	1044891	1071	546	231
SerUGA-15	6	1097223	1097304	2184	7320	314
SerUGA-9	6	1124940	1125021	0	776	62
SerUGA-14	6	1129630	1129711	0	0	0
SerUGA-13	6	1133095	1133176	25	23115	862
ProUGG-16	6	1250562	1250632	0	0	0
ProUGG-15	6	1299996	1300066	2	0	4
ValAAC-21	6	1321993	1322066	0	69	0
IleAAU-12	6	1374085	1374157	0	0	0
GlnUUG-13	6	1453608	1453680	0	1	7
AlaAGC-15	6	1529428	1529500	0	0	0
CysGCA-8	6	1664089	1664160	0	232	21
LysUUU-23	6	1703275	1703347	21	2159	1137
SerUGA-10	6	1758891	1758972	0	1274	113
SerUGA-12	6	1761326	1761407	0	22450	1684
LeuUAA-13	6	1776201	1776283	3522	160	59
LysUUU-20	6	1794634	1794706	1	7375	2384
AsnGUU-19	6	1801506	1801578	0	71	46
AsnGUU-16	6	1833377	1833449	0	24	34
GlyGCC-15	6	1836361	1836431	0	0	0
GlyGCC-18	6	1837565	1837635	358	0	193
HisGUG-10	6	1837996	1838066	2	7	3
ProAGG-1	6	1869424	1869494	0	0	0
LeuUAA-17	6	1870453	1870535	116	4221	1857
GlyGCC-16	6	1912886	1912956	17	5	6
GlyGCC-17	6	1927240	1927310	0	0	12
MetCAU-17	6	1948217	1948288	0	0	480
LeuAAG-8	6	2082530	2082611	0	0	12
TyrGUA-9	6	2088994	2089076	0	14	1138
GluUUC-16	6	2120506	2120577	8	155	7
TyrGUA-10	6	2176055	2176137	1	82	358
CysGCA-7	6	2258615	2258686	11067	196	20
GlnUUG-14	6	2286132	2286204	0	1	4
SerAGA-9	6	2340655	2340736	5165	4920	286
SerGCU-12	6	2347717	2347797	12	2212	240
GluCUC-2	6	2365116	2365187	7	1	1
GluCUC-3	6	2478517	2478588	52	220	23
AlaAGC-14	6	2479676	2479748	4	2030	359
TyrGUA-13	6	2597197	2597279	0	16277	194964
ThrAGU-18	6	2598517	2598588	0	1571	204
AlaAGC-13	6	2624351	2624423	0	258	23
LeuUAA-16	6	2624493	2624575	13	156	38
AlaAGC-12	6	2625762	2625834	0	1509	42
LeuAAG-9	6	2728442	2728523	0	0	90
GluUUC-20	6	2730062	2730133	78	48	6

ValUAC-7	6	2768404	2768477	1791	2929	555
IleUAU-4	6	2770337	2770427	3	1	0
IleUAU-3	6	2771961	2772051	0	4	4
AspGUC-21	6	2777486	2777557	3	31	1
IleAAU-13	6	2784596	2784668	0	8	6
LeuAAG-10	6	2836863	2836944	9	0	455
MetCAU-16	6	2840202	2840274	0	54	21
HisGUG-8	6	2840392	2840462	19	2	1
IleAAU-16	6	2936034	2936106	11	10	1
LysCUU-10	6	2954533	2954605	0	0	0
LysCUU-9	6	2956996	2957068	0	0	0
LysCUU-8	6	2985396	2985468	0	0	14
AspGUC-22	6	3008610	3008681	8	368	93
GluUUC-17	6	3053148	3053219	19	11054	335
TyrGUA-11	6	3068583	3068665	7	10	156
GluUUC-19	6	3069439	3069510	0	126	30
AlaAGC-11	6	3132339	3132411	133	3367	118
IleAAU-14	6	3133759	3133831	2	0	0
LysUUU-22	6	3137397	3137469	38	1536	148
LysUUU-21	6	3141444	3141516	7	142	91
HisGUG-9	6	3173627	3173697	4	32	5
AsnGUU-17	6	3182148	3182220	0	534	558
AsnGUU-18	6	3235027	3235099	0	384	365
GluUUC-18	6	3291634	3291705	6	1077	391
IleUAU-2	6	3299859	3299949	4901	327	3
IleUAU-1	6	3312699	3312789	84	1060	80
SerUGA-11	6	3325552	3325633	9	2786	104
PheGAA-14	6	3387585	3387658	0	569	98
LeuAAG-11	6	3407225	3407306	0	3	237
TyrGUA-12	6	3430565	3430646	0	0	9
LeuUAA-15	6	3431954	3432036	229	19	10
LeuUAA-14	6	3448553	3448635	0	5844	2924
IleAAU-15	6	3534943	3535015	76	50	14
PheGAA-15	6	3574106	3574179	0	2827	911
ArgCCU-1	6	3587001	3587074	221	1571	11
